# Nitrogen Reduction Combined with Calcium-Based Amendments Regulates Soil Acidification Buffering and Maize Productivity in Acidified Croplands of Northeast China

**DOI:** 10.3390/plants15203116

**Published:** 2026-10-12

**Authors:** Yiting Liu, Letian Jin, Na Yuan, Mengmeng Bao, Fengzhuo Geng, Dongran Liu, Jiachen Tong, Iseult Lynch, Xiuli Dang

**Affiliations:** 1 National Engineering Research Center for Efficient Utilization of Soil and Fertilizer Resources, Key Laboratory of Arable Land Conservation in Northeast China, Ministry of Agriculture and Rural Affairs, College of Land and Environment, Shenyang Agricultural University, Shenyang 110866, China; 2024220437@stu.syau.edu.cn (Y.L.); jlt2000@syau.edu.cn (L.J.); yn0121@stu.syau.edu.cn (N.Y.); bmm121@stu.syau.edu.cn (M.B.); 2024220452@stu.syau.edu.cn (F.G.); imnot1iudong@syau.edu.cn (D.L.); 2025240676@stu.syau.edu.cn (J.T.); 2 School of Geography, Earth and Environmental Sciences, University of Birmingham, Birmingham B15 2TT, UK; i.lynch@bham.ac.uk

**Keywords:** acidified soil, nitrogen reduction, calcium-based amendments, dolomite, soil buffering capacity, maize yield

## Abstract

Excessive nitrogen fertilization accelerates soil acidification and threatens soil fertility and crop productivity, highlighting the need for management strategies that reduce nitrogen inputs while maintaining soil function and crop production. This study investigated the effects of nitrogen reduction combined with calcium-based amendments on soil acidity, nutrient availability, and maize productivity in an acidified aquic brown soil. A field experiment was conducted with three nitrogen-reduction levels (10%, 20%, and 30%) and four amendment treatments (no amendment, dolomite, quicklime, and fused calcium–magnesium phosphate fertilizer). The results showed that, under non-amended conditions, greater nitrogen reduction resulted in higher soil pH and measured total exchangeable base cations (TEBC), whereas nitrogen-reduction level had no significant effect on soil pH buffering capacity (pHBC). Among the calcium-based amendments, dolomite and quicklime increased pHBC by 25.3% and 21.4%, respectively, relative to the non-amended treatment, while dolomite generally showed higher measured concentrations of exchangeable Ca^2+^ and Mg^2+^ and higher TEBC. Amendment effects on nutrient availability differed among treatments: fused calcium–magnesium phosphate fertilizer produced the highest available phosphorus, whereas dolomite increased alkali-hydrolyzable nitrogen and generally resulted in higher NH_4_^+^-N and available potassium. Maize grain yield was maintained when nitrogen reduction increased from 10% to 20% but declined significantly under 30% nitrogen reduction. Across nitrogen-reduction levels, dolomite was associated with the highest grain yield, exceeding the non-amended and quicklime treatments by 4.1% and 5.4%, respectively. Overall, moderate nitrogen reduction maintained maize productivity, while dolomite showed favorable responses across multiple indicators of soil acidity, exchangeable base-cation status, nutrient availability, and grain yield. These findings highlight the importance of coordinating nitrogen-reduction intensity with amendment-specific effects to balance soil amelioration and maize productivity in acidified croplands.

## 1. Introduction

Soil acidification has emerged as a major constraint to sustainable agricultural production worldwide, particularly in intensively managed croplands subjected to long-term chemical fertilizer inputs [1,2]. Among various anthropogenic drivers, excessive nitrogen fertilization is considered a primary contributor to soil acidification because enhanced nitrification accelerates proton production and promotes the depletion of exchangeable base cations through leaching and crop removal processes [3,4]. Progressive soil acidification can impair nutrient availability, alter soil chemical properties, and ultimately reduce crop productivity [1,5]. Therefore, understanding the mechanisms underlying soil acidification amelioration is essential for developing sustainable management strategies in agricultural systems.

Reducing excessive nitrogen inputs can alleviate fertilizer-induced acidification by decreasing the proton production associated with nitrogen transformation processes [6,7]. However, excessive nitrogen reduction may compromise crop productivity due to insufficient nutrient supply, highlighting the importance of balancing reduced fertilizer inputs with crop nutrient requirements and nutrient-use efficiency [6,7]. In addition, reducing nitrogen inputs alone may have limited capacity to rapidly ameliorate soil acidity. Calcium-based amendments, including dolomite, quicklime, and fused calcium–magnesium phosphate fertilizer (CMP), have been widely applied to improve acidic soils through acidity neutralization and the replenishment of depleted base cations [8,9]. However, these amendments differ substantially in their chemical reactivity and nutrient supply capacity, resulting in variable effects on soil chemical properties, nutrient availability, and crop responses [8,10]. Therefore, integrating appropriate nitrogen reduction with suitable calcium-based amendments may provide a promising strategy to balance soil acidification amelioration and crop productivity maintenance.

Previous studies on acidic soil remediation have primarily focused on changes in soil pH. However, soil pH mainly reflects the current acidity status and does not fully represent the ability of soil to resist future acid inputs [11]. Soil pH buffering capacity (pHBC), which describes the resistance of soil pH to external acidification, provides a more comprehensive indicator of soil acidification resistance [4,12,13]. Enhancing pHBC requires not only neutralization of existing acidity but also replenishment of depleted exchangeable base cations and improvement of soil nutrient availability [4,11]. Despite its importance, the extent to which nitrogen management and calcium-based amendments interactively regulate soil pHBC, exchangeable base cations, and associated changes in soil fertility remains insufficiently understood [4,14].

Northeast China is one of the major maize-producing regions worldwide, where intensive nitrogen fertilization and long-term cultivation have increased the risk of soil acidification. Maintaining soil fertility and yield stability is essential for sustainable production in this region [4,11]. Therefore, developing management strategies that enhance soil acidification resistance while sustaining crop productivity is of particular importance for acidic maize soils.

To address this knowledge gap, we conducted a field experiment to investigate how different nitrogen-reduction levels combined with calcium-based amendments (dolomite, quicklime, and fused calcium–magnesium phosphate fertilizer) regulate soil acidity characteristics, nutrient availability, and maize productivity in an acidic maize soil. Specifically, this study aimed to (i) determine the effects of nitrogen reduction and calcium-based amendments on soil acidity indicators, including soil pH, pH buffering capacity, and exchangeable base-cation pools; (ii) evaluate their impacts on soil nutrient availability and maize yield formation; and (iii) explore the relationships between soil acidification amelioration and maize productivity responses. We hypothesized that combining nitrogen reduction with calcium-based amendments would enhance soil acidification resistance by improving soil buffering capacity, increasing exchangeable base-cation availability, and promoting nutrient availability, while the magnitude of these effects would vary among amendment types.

## 2. Results

### 2.1. Regulation of Soil Acidity Status and Buffering Capacity by Nitrogen-Reduction Level and Calcium-Based Amendments

#### 2.1.1. Effects of Nitrogen-Reduction Level and Calcium-Based Amendments on Soil pH

**Figure 1:**
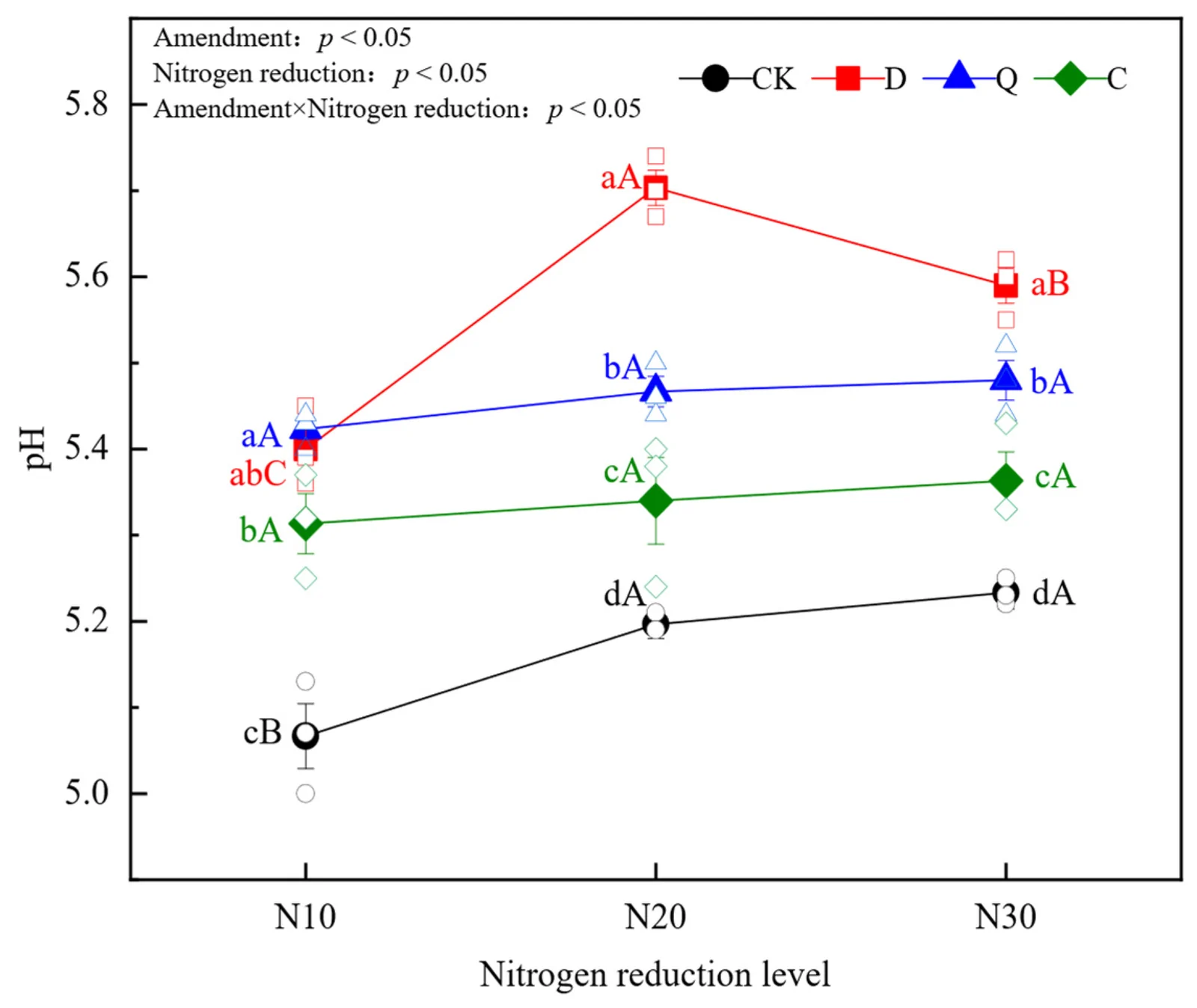
Soil pH under nitrogen reduction and calcium-based amendment treatments. Values are means ± SE (*n* = 3). Different lowercase letters indicate significant differences among amendment treatments within the same nitrogen-reduction level, whereas different uppercase letters indicate significant differences among nitrogen-reduction levels within the same amendment treatment (Tukey’s HSD, *p* < 0.05). N10, N20, and N30 denote 10%, 20%, and 30% nitrogen reduction, respectively; CK, D, Q, and C denote no amendment, dolomite, quicklime, and fused calcium–magnesium phosphate fertilizer, respectively.

Soil pH was significantly affected by nitrogen-reduction level, calcium-based amendment, and their interaction (Figure 1), indicating that the response of soil pH to nitrogen reduction varied with amendment treatment. Under the non-amended condition, increasing nitrogen reduction from 10% to 20% significantly increased soil pH from 5.07 to 5.20 (*p* < 0.05), whereas increasing nitrogen reduction to 30% did not result in a further significant change (*p* > 0.05). Dolomite showed a distinct response to nitrogen reduction, with soil pH increasing significantly from 5.40 under N10D to 5.70 under N20D (*p* < 0.05), followed by a significant decrease to 5.59 under N30D (*p* < 0.05). In contrast, soil pH remained statistically unchanged across the three nitrogen-reduction levels under quicklime and calcium–magnesium phosphate fertilizer application (*p* > 0.05).

Amendment effects became more distinct as nitrogen reduction increased. Under N10, quicklime resulted in significantly higher soil pH than CMP, whereas dolomite did not differ significantly from either amendment; all three amendments significantly increased soil pH relative to CK (*p* < 0.05). Under both N20 and N30, soil pH consistently followed the order D > Q > C > CK, with significant differences among all four amendment treatments (*p* < 0.05). The highest soil pH was observed under N20D (5.70), which was 0.23 and 0.36 pH units higher than those under N20Q and N20C, respectively (*p* < 0.05).

#### 2.1.2. Response of Soil pH Buffering Capacity to Nitrogen-Reduction Level and Calcium-Based Amendments

**Figure 2:**
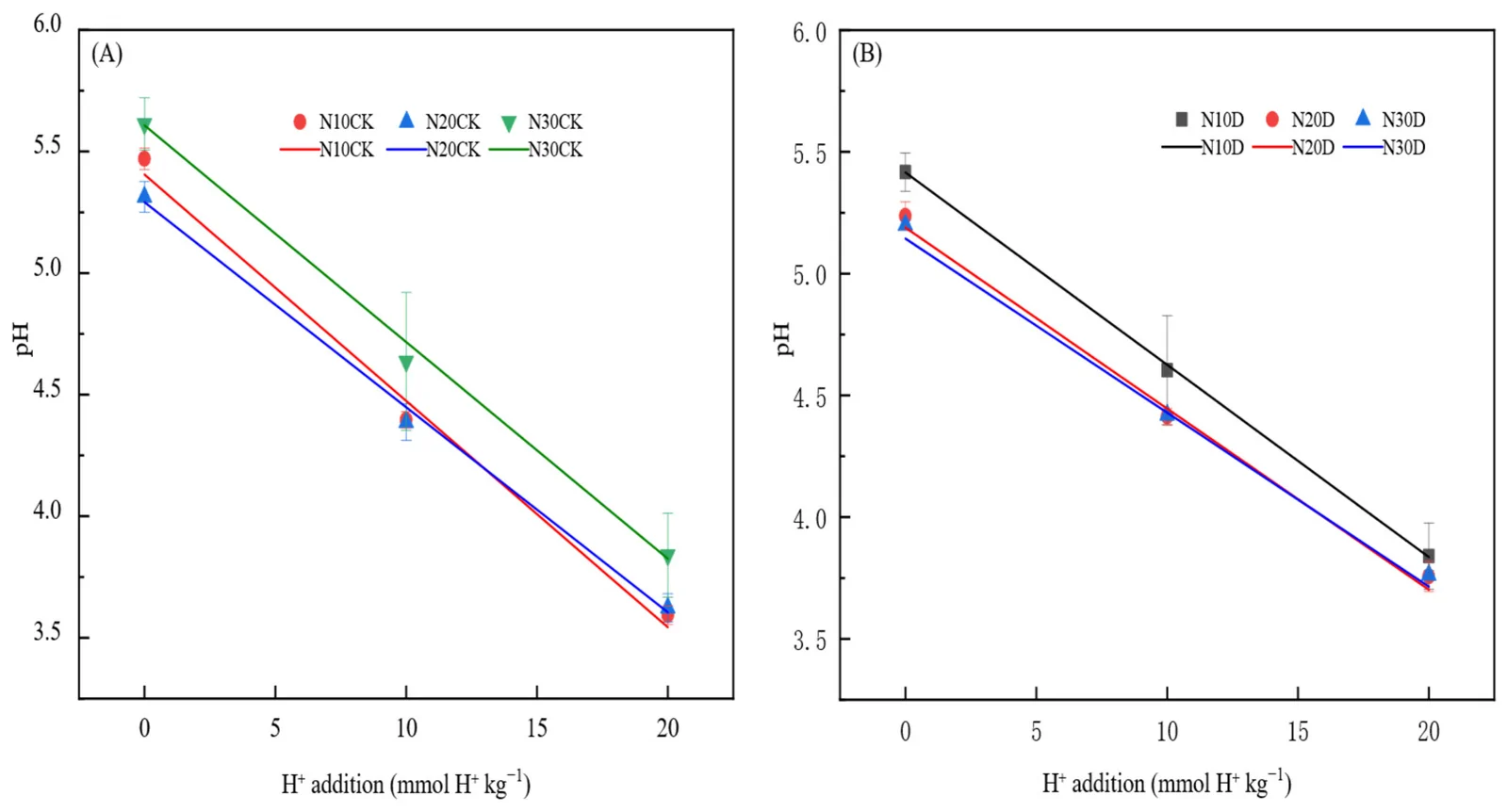

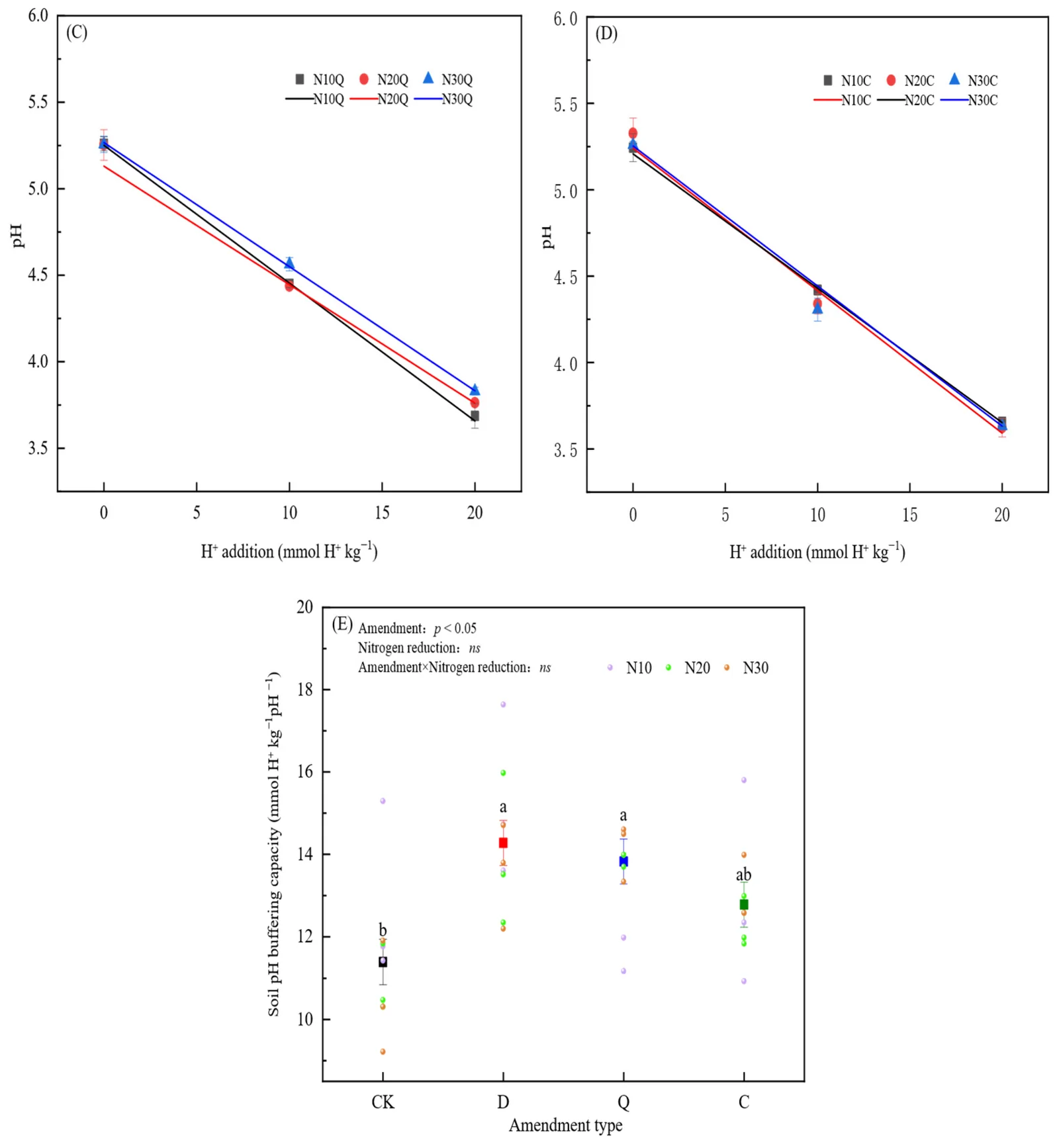
Soil pH buffering capacity (pHBC) under nitrogen reduction and calcium-based amendment treatments. (**A**) Soil pH response to acid addition under CK; (**B**) soil pH response under dolomite; (**C**) soil pH response under quicklime; (**D**) soil pH response under fused calcium–magnesium phosphate fertilizer; (**E**) effects of amendment treatments on pHBC across nitrogen-reduction levels. Values in (**A**–**D**) are means ± SE (*n* = 3). In (**E**), large symbols and error bars represent estimated marginal means ± model SE, whereas small symbols represent individual observations. Different lowercase letters indicate significant differences among amendment treatments according to Tukey’s test (*p* < 0.05). *p* < 0.05 indicates a significant effect, and ns indicates a non-significant effect. N10, N20, and N30 denote 10%, 20%, and 30% nitrogen reduction, respectively; CK, D, Q, and C denote no amendment, dolomite, quicklime, and calcium–magnesium phosphate fertilizer, respectively.

Soil pH declined progressively with increasing acid addition across all treatments over the complete titration range (Figure S1). Within the low-acid-addition range used for pHBC estimation, soil pH also showed a consistent decreasing response to acid addition across the 12 treatment combinations (Figure 2A–D). The rate of pH decline varied among amendment treatments, with dolomite and quicklime generally showing a smaller pH decrease than the non-amended treatment, consistent with their greater resistance to acid addition. The linear regressions within this range yielded *R*^2^ values ranging from 0.897 to 1.000. Plot-level regression parameters and the corresponding pHBC values are provided in Table S1.

Soil pHBC was significantly affected by calcium-based amendment (*p* < 0.05), whereas nitrogen-reduction level and the interaction between nitrogen-reduction level and amendment treatment had no significant effects (Figure 2E). Relative to CK, dolomite and quicklime significantly increased pHBC by 25.3% and 21.4%, respectively. CMP showed an intermediate response and did not differ significantly from CK, dolomite, or quicklime.

### 2.2. Responses of Exchangeable Base-Cation Composition and Pools to Nitrogen-Reduction Level and Calcium-Based Amendments

**Figure 3:**
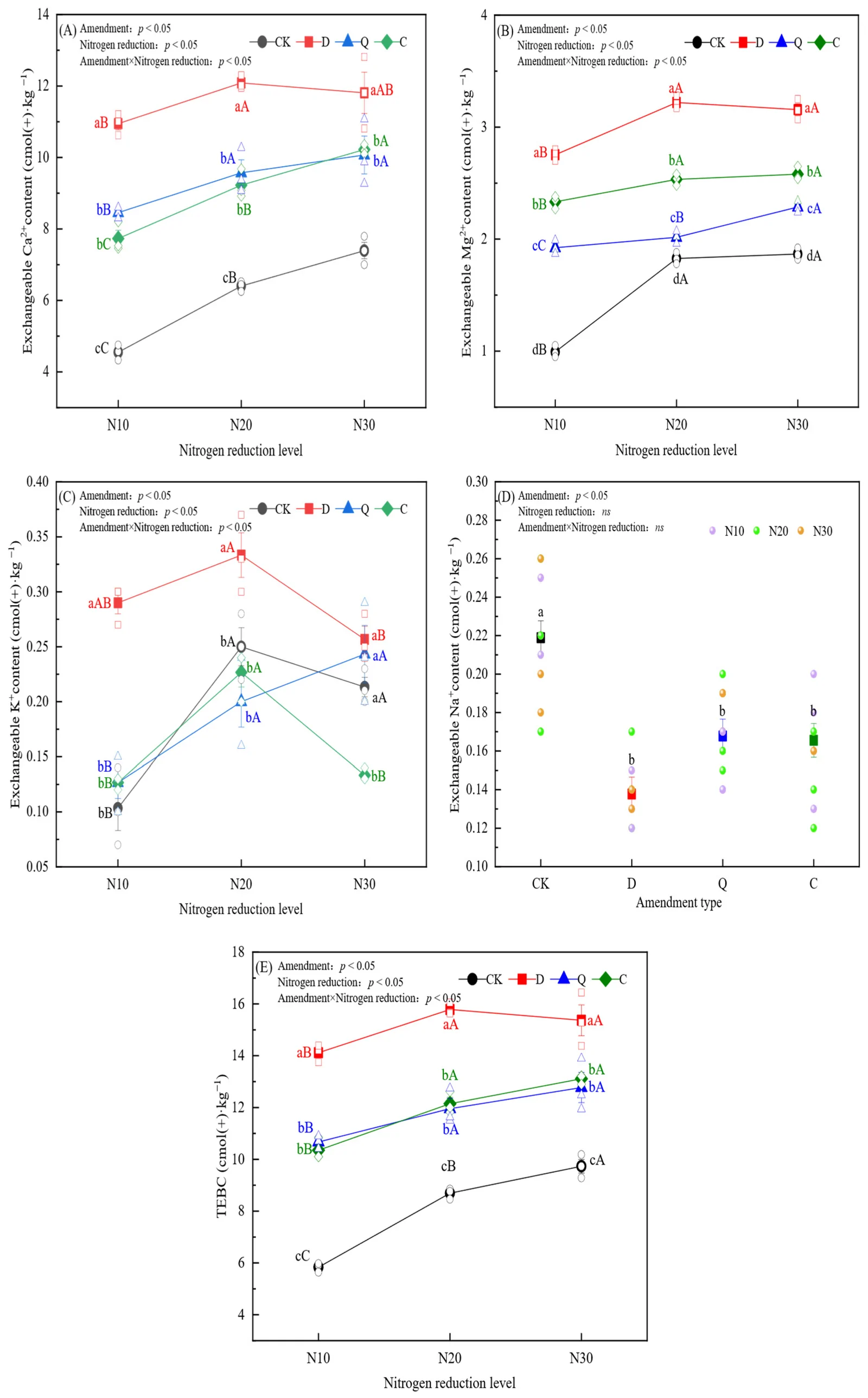
Exchangeable base cations and total exchangeable base cations (TEBC) under nitrogen reduction and calcium-based amendment treatments. (**A**) Exchangeable Ca^2+^; (**B**) exchangeable Mg^2+^; (**C**) exchangeable K^+^; (**D**) exchangeable Na^+^; (**E**) TEBC. Values in (**A**–**C**) and (**E**) are means ± SE (*n* = 3); different lowercase and uppercase letters indicate significant differences among amendments within each nitrogen-reduction level and among nitrogen-reduction levels within each amendment, respectively (Tukey’s HSD, *p* < 0.05). In (**D**), large symbols and error bars represent estimated marginal means ± model SE, whereas small symbols represent individual observations; different lowercase letters indicate significant amendment effects (Tukey’s HSD, *p* < 0.05). ns, not significant. N10, N20, and N30 denote 10%, 20%, and 30% reductions, respectively; CK, D, Q, and C denote no amendment, dolomite, quicklime, and fused calcium–magnesium phosphate fertilizer, respectively.

Exchangeable Ca^2+^ and Mg^2+^ were significantly affected by nitrogen reduction, amendment, and their interaction (Figure 3A,B). Under the non-amended condition, increasing nitrogen reduction from 10% to 20% significantly increased both Ca^2+^ and Mg^2+^ (*p* < 0.05), whereas a further increase to 30% produced no significant change (*p* > 0.05). Dolomite consistently produced the highest exchangeable Ca^2+^ concentration at each nitrogen-reduction level, exceeding the corresponding CK by 140.7%, 88.9%, and 59.8% under N10, N20, and N30, respectively (*p* < 0.05). Exchangeable Mg^2+^ consistently followed the order D > C > Q > CK across all three nitrogen-reduction levels (*p* < 0.05), with dolomite increasing Mg^2+^ by 69.0–177.2% relative to CK.

Exchangeable K^+^ was also significantly affected by nitrogen reduction, amendment, and their interaction (Figure 3C). Dolomite produced the highest K^+^ concentrations under N10 and N20 (*p* < 0.05), whereas under N30, K^+^ did not differ significantly among CK, D, and Q (*p* > 0.05), with CMP showing a significantly lower concentration (*p* < 0.05). In contrast, exchangeable Na^+^ was affected only by amendment (Figure 3D), with D, Q, and C showing significantly lower concentrations than CK (*p* < 0.05). Plot-level values of exchangeable Ca^2+^, Mg^2+^, K^+^, and Na^+^ and the corresponding TEBC are provided in Table S2. The normalized patterns of the four exchangeable base cations across treatment combinations are shown in Figure S2.

TEBC was significantly affected by nitrogen reduction, amendment, and their interaction (Figure 3E). Dolomite consistently exhibited the highest measured TEBC at each nitrogen-reduction level, exceeding the corresponding CK by 141.8%, 81.6%, and 58.0% under N10, N20, and N30, respectively (*p* < 0.05). Post-harvest CEC varied among treatments and was numerically highest under dolomite at each nitrogen-reduction level, ranging from 28.60 to 33.19 cmol(+)·kg^−1^. Exchangeable acidity was generally lower in the amended treatments than in the corresponding non-amended treatments, with the lowest values observed under quicklime. Treatment means of TEBC, CEC, exchangeable acidity, and base saturation are provided in Table S3.

### 2.3. Effects of Nitrogen-Reduction Level and Calcium-Based Amendments on Soil Nutrient Availability

#### 2.3.1. Responses of Soil Organic Matter to Nitrogen-Reduction Level and Calcium-Based Amendments

**Figure 4:**
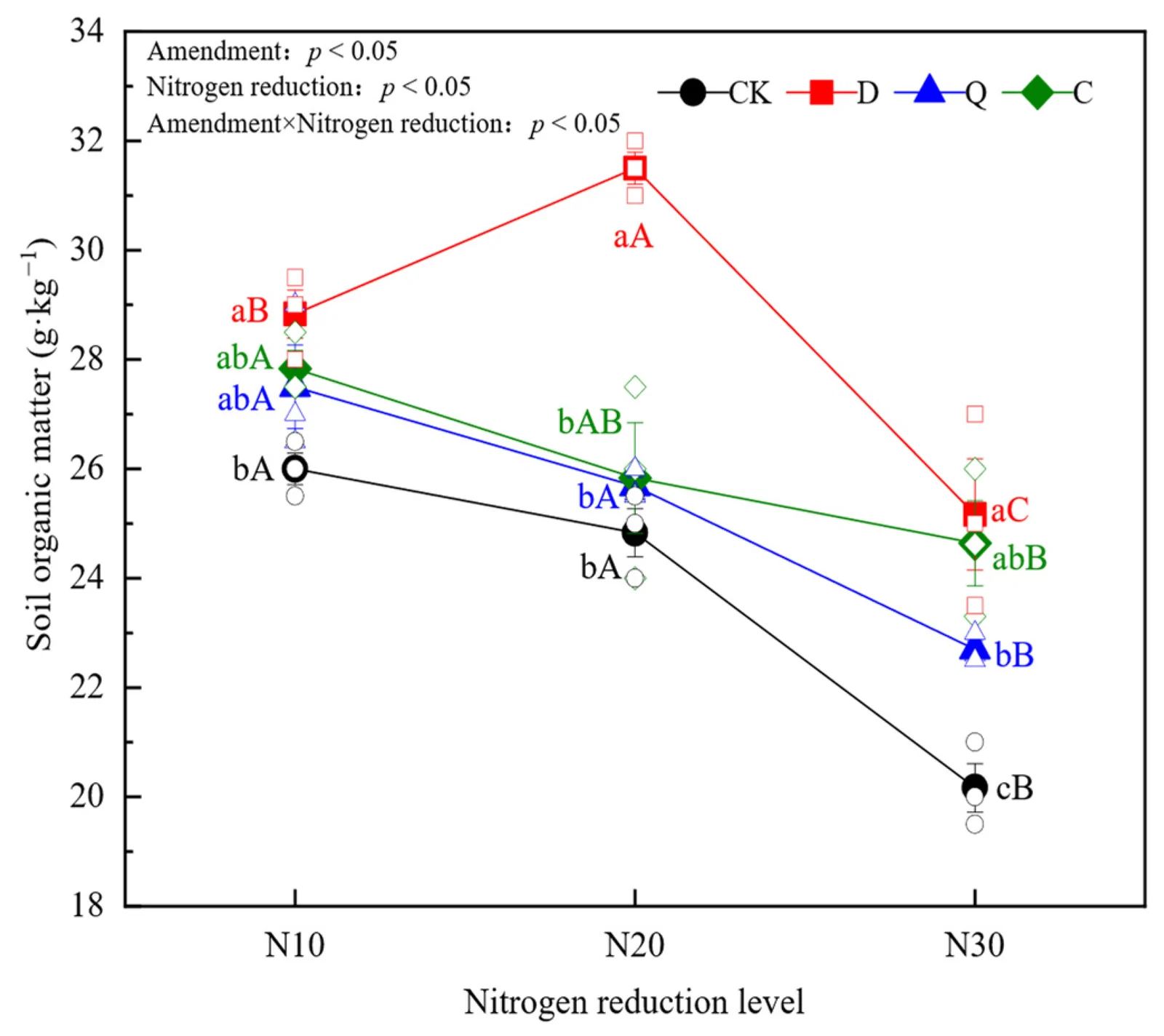
Soil organic matter under nitrogen reduction and calcium-based amendment treatments. Large symbols and error bars represent means ± SE (*n* = 3), whereas small symbols represent individual observations. Different lowercase and uppercase letters indicate significant differences among amendment treatments within the same nitrogen-reduction level and among nitrogen-reduction levels within the same amendment treatment, respectively (Tukey’s HSD, *p* < 0.05). N10, N20, and N30 denote 10%, 20%, and 30% nitrogen reduction, respectively; CK, D, Q, and C denote no amendment, dolomite, quicklime, and fused calcium–magnesium phosphate fertilizer, respectively.

Soil organic matter (SOM) was significantly affected by nitrogen-reduction level, calcium-based amendment, and their interaction (Figure 4). Under the non-amended condition, SOM remained unchanged from N10 to N20 (*p* > 0.05) but decreased significantly under N30 (*p* < 0.05). Amendment application modified this response, with dolomite showing the most pronounced effect under N20, where SOM was significantly higher than under the other three amendment treatments (*p* < 0.05). Within the dolomite treatments, SOM increased significantly from N10 to N20 and subsequently decreased under N30 (*p* < 0.05). Under N30, all three amendments resulted in significantly higher SOM than CK (*p* < 0.05). Dolomite also maintained significantly higher SOM than quicklime (*p* < 0.05), whereas CMP did not differ significantly from either amendment (*p* > 0.05).

#### 2.3.2. Response of Soil Nitrogen Availability and Inorganic Nitrogen Composition to Nitrogen-Reduction Level and Calcium-Based Amendments

**Figure 5:**
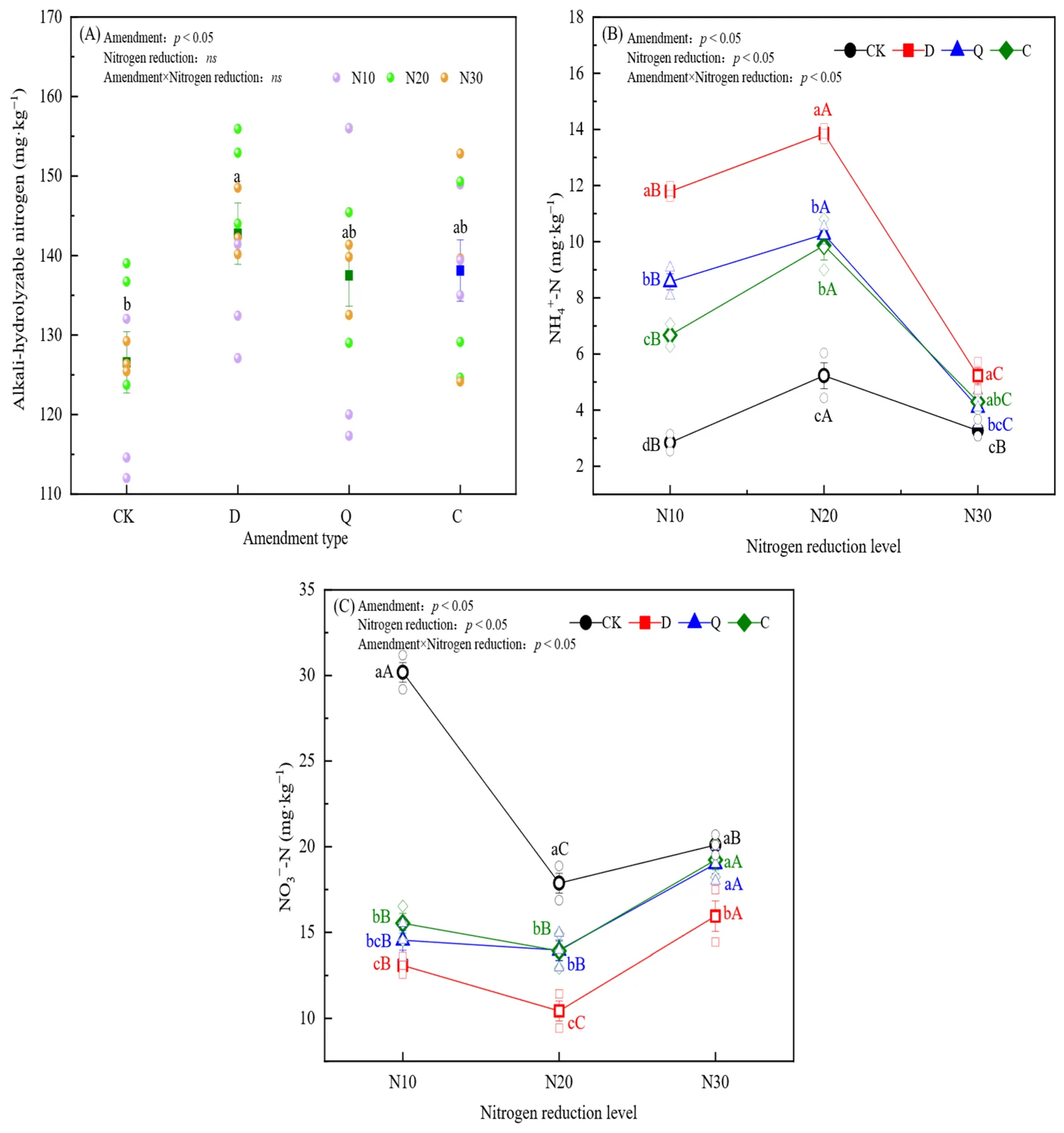
Soil nitrogen availability under nitrogen reduction and calcium-based amendment treatments. (**A**) Alkali-hydrolyzable nitrogen (AHN); (**B**) NH_4_^+^-N; (**C**) NO_3_^−^-N. In (**A**), large symbols and error bars represent estimated marginal means ± model SE, whereas small symbols represent individual observations; different lowercase letters indicate significant differences among amendment treatments (Tukey’s HSD, *p* < 0.05). In (**B**,**C**), values are means ± SE (*n* = 3); different lowercase and uppercase letters indicate significant differences among amendment treatments within each nitrogen-reduction level and among nitrogen-reduction levels within each amendment treatment, respectively (Tukey’s HSD, *p* < 0.05). ns, not significant. N10, N20, and N30 denote 10%, 20%, and 30% nitrogen reduction, respectively; CK, D, Q, and C represent no amendment, dolomite, quicklime, and fused calcium–magnesium phosphate fertilizer, respectively.

Alkali-hydrolyzable nitrogen (AHN) was significantly affected by amendment treatment, whereas neither nitrogen reduction nor the interaction between nitrogen reduction and amendment treatment significantly affected AHN (Figure 5A). Across nitrogen-reduction levels, dolomite significantly increased AHN by 12.8% relative to CK (*p* < 0.05), whereas quicklime and CMP did not differ significantly from either CK or dolomite (*p* > 0.05).

NH_4_^+^-N was significantly affected by nitrogen reduction, amendment treatment, and their interaction (Figure 5B). Under the non-amended condition, NH_4_^+^-N increased significantly from N10 to N20 and subsequently decreased significantly under N30 (*p* < 0.05). Amendment application markedly increased NH_4_^+^-N, particularly under N10, where dolomite, quicklime, and CMP increased NH_4_^+^-N by 315.1%, 201.8%, and 134.9%, respectively, relative to CK (*p* < 0.05). Under N20, dolomite produced the highest NH_4_^+^-N concentration, while quicklime and CMP showed comparable responses and both significantly exceeded CK (*p* < 0.05). Under N30, dolomite and CMP maintained significantly higher NH_4_^+^-N concentrations than CK (*p* < 0.05).

NO_3_^−^-N showed an opposite response, with significant effects of nitrogen reduction, amendment treatment, and their interaction (Figure 5C). Under the non-amended condition, NO_3_^−^-N decreased significantly by 40.8% from N10 to N20 and subsequently increased significantly under N30, although it remained 33.4% lower than under N10 (*p* < 0.05). Dolomite significantly reduced NO_3_^−^-N relative to CK at all three nitrogen-reduction levels, by 56.6%, 41.6%, and 20.6% under N10, N20, and N30, respectively (*p* < 0.05). Quicklime and CMP also significantly reduced NO_3_^−^-N under N10, whereas their effects became less pronounced at the higher nitrogen-reduction levels.

#### 2.3.3. Responses of Available Phosphorus and Potassium to Nitrogen-Reduction Level and Calcium-Based Amendments

**Figure 6:**
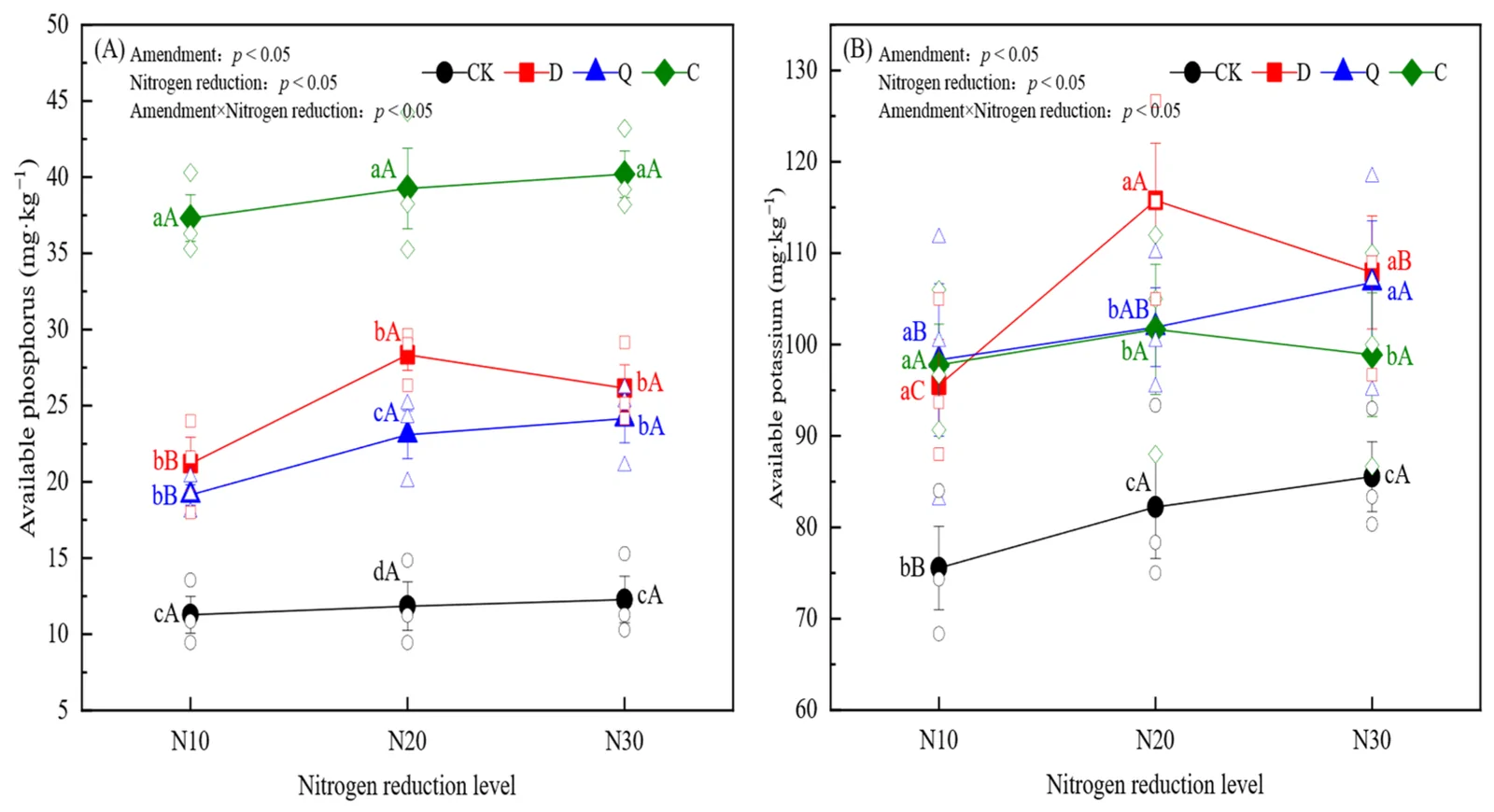
Soil available phosphorus and potassium under nitrogen reduction and calcium-based amendment treatments. (**A**) Available phosphorus; (**B**) available potassium. Values are means ± SE (*n* = 3); different lowercase and uppercase letters indicate significant differences among amendment treatments within each nitrogen-reduction level and among nitrogen-reduction levels within each amendment treatment, respectively (Tukey’s HSD, *p* < 0.05). ns, not significant. N10, N20, and N30 denote 10%, 20%, and 30% nitrogen reduction, respectively; CK, D, Q, and C denote no amendment, dolomite, quicklime, and fused calcium–magnesium phosphate fertilizer, respectively.

Available phosphorus was significantly affected by nitrogen reduction, amendment treatment, and their interaction (Figure 6A). Under the non-amended condition, available phosphorus remained unchanged across the three nitrogen-reduction levels (*p* > 0.05), indicating a limited response to nitrogen reduction alone. In contrast, amendment application substantially increased available phosphorus, with CMP consistently producing the highest concentrations across all nitrogen-reduction levels and increasing available phosphorus by 227.6–231.5% relative to the corresponding CK treatments (*p* < 0.05). The effects of dolomite and quicklime were smaller and depended on nitrogen-reduction level.

Available potassium was also significantly affected by nitrogen reduction, amendment treatment, and their interaction (Figure 6B). Under the non-amended condition, available potassium increased significantly from N10 to N20 and remained at a comparable level under N30. All three amendments significantly increased available potassium relative to CK across the nitrogen-reduction levels (*p* < 0.05), but their relative effects varied with nitrogen reduction. The clearest differentiation occurred under N20, where dolomite produced the highest available potassium, exceeding quicklime and CMP by 13.6% and 13.9%, respectively (*p* < 0.05). Under N30, dolomite and quicklime maintained significantly higher available potassium than CMP and CK (*p* < 0.05).

### 2.4. Responses of Maize Productivity to Nitrogen-Reduction Level and Calcium-Based Amendments

**Figure 7:**
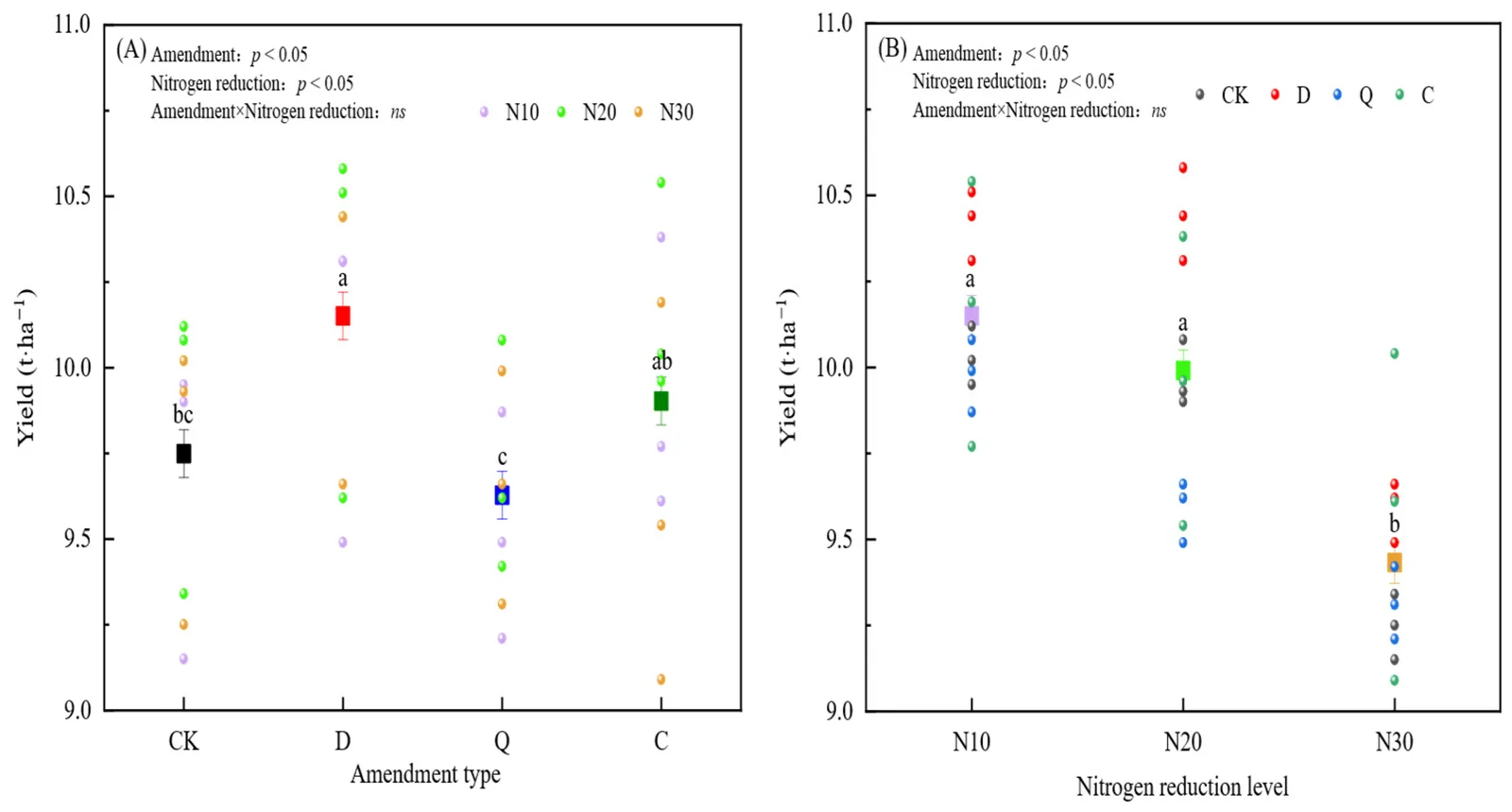
Maize grain yield under nitrogen reduction and calcium-based amendment treatments. (**A**) Amendment main effect; (**B**) nitrogen-reduction main effect. Large symbols and error bars represent estimated marginal means ± model SE, and small symbols represent individual observations. Different lowercase letters indicate significant differences among amendment treatments in (**A**) and among nitrogen-reduction levels in (**B**) (Tukey’s HSD, *p* < 0.05). ns, not significant. N10, N20, and N30 denote 10%, 20%, and 30% nitrogen reduction, respectively; CK, D, Q, and C represent no amendment, dolomite, quicklime, and fused calcium–magnesium phosphate fertilizer, respectively.

Maize grain yield was significantly affected by nitrogen reduction and amendment treatment, whereas their interaction had no significant effect (Figure 7). Across nitrogen-reduction levels, dolomite produced the highest grain yield, exceeding CK and quicklime by 4.1% and 5.4%, respectively (*p* < 0.05), but did not differ significantly from CMP (*p* > 0.05). Across amendment treatments, grain yield remained comparable between N10 and N20 (*p* > 0.05) but decreased significantly under N30, by 7.1% and 5.6% relative to N10 and N20, respectively (*p* < 0.05). These results indicate that yield was maintained with nitrogen reduction up to 20%, whereas a 30% reduction resulted in a significant yield penalty.

### 2.5. Integrated Responses of Soil Acidity Amelioration and Maize Productivity Revealed by PCA

**Figure 8:**
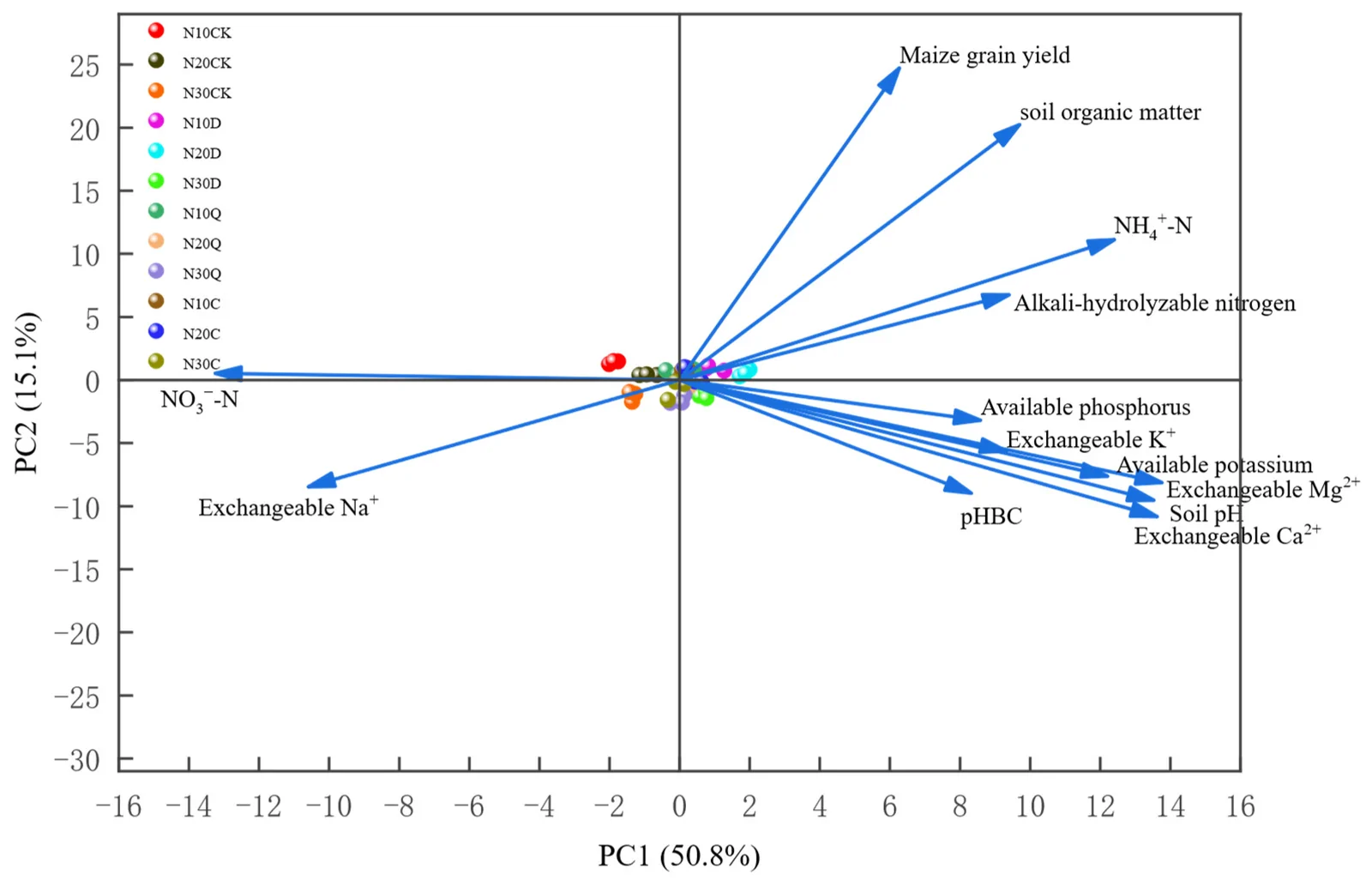
Principal component analysis (PCA) biplot of soil properties and maize grain yield under nitrogen reduction and calcium-based amendment treatments. Points represent PCA scores of individual experimental replicates from the 12 treatment combinations, and arrows represent variable loadings. N10, N20, and N30 denote 10%, 20%, and 30% nitrogen reduction, respectively; CK, D, Q, and C denote no amendment, dolomite, quicklime, and fused calcium–magnesium phosphate fertilizer, respectively.

Principal component analysis (PCA) revealed distinct multivariate responses of soil properties and maize productivity to nitrogen reduction and amendment treatments (Figure 8). The first three principal components explained 75.4% of the total variance (Table S4), with PC1 and PC2 accounting for 50.8% and 15.1%, respectively. PC1 primarily represented variation in soil acidity and exchangeable base-cation status, with soil pH and exchangeable Ca^2+^ and Mg^2+^ showing strong positive loadings, whereas PC2 was mainly associated with maize grain yield and soil organic matter (Table S5).

The treatments showed contrasting distributions along PC1, particularly between dolomite-amended and non-amended treatments. Dolomite-amended treatments were clearly shifted toward positive PC1 scores, with N20D showing the strongest positive shift in the direction of soil pH and exchangeable Ca^2+^ and Mg^2+^. Quicklime- and CMP-amended treatments were also predominantly located on the positive side of PC1 but showed less pronounced shifts than dolomite, whereas the non-amended treatments were mainly distributed on the negative side.

The PCA loading pattern further showed that maize grain yield was closely aligned with soil organic matter, whereas soil pH was closely aligned with exchangeable Ca^2+^ and Mg^2+^. Consistent with these relationships, maize grain yield was strongly correlated with soil organic matter (r = 0.79), while soil pH was strongly correlated with exchangeable Ca^2+^ (r = 0.90) and Mg^2+^ (r = 0.82; Table S6).

## 3. Discussion

### 3.1. Calcium-Based Amendments Improved Soil pH Buffering and Exchangeable Base-Cation Status

Our results showed that increasing the degree of nitrogen reduction within the 10–30% range was associated with higher soil pH under non-amended conditions, consistent with reduced acidification pressure at lower nitrogen inputs. The concurrent increase in TEBC with increasing nitrogen reduction should not, however, be interpreted as direct replenishment of the soil base-cation pool. Reduced nitrogen input may decrease proton production associated with nitrogen transformations, thereby alleviating acidification pressure and potentially contributing to the observed differences in post-harvest exchangeable base-cation status. The absence of a significant response in pHBC indicates that reducing nitrogen input alone did not measurably alter soil buffering capacity within the 10–30% reduction range during a single growing season.

Calcium-based amendments produced a more pronounced improvement in soil acidity-related properties and exchangeable base-cation status than nitrogen reduction alone. Both dolomite and quicklime significantly increased pHBC, indicating greater resistance to acid addition, while dolomite was additionally associated with greater post-harvest exchangeable Ca^2+^ and Mg^2+^ concentrations and TEBC. Previous studies have shown that liming materials alleviate soil acidity through proton consumption and increase exchangeable Ca^2+^ and Mg^2+^ in acidic soils [14]. In the present study, dolomite was also associated with higher post-harvest CEC, which may reflect changes in pH-dependent surface charge and soil exchange properties. In acidic soils, an increase in pH promotes deprotonation of pH-dependent functional groups on soil organic matter and mineral surfaces, increasing negative surface charge and potentially contributing to higher CEC. Such pH-dependent changes may influence cation retention and contribute to differences in measured CEC [15]. The substantial differences between initial and post-harvest CEC values may also partly reflect inherent spatial heterogeneity in soil exchange properties, as the initial soil was characterized using a field-composite sample.

In addition, NH_4_OAc extraction can partially dissolve carbonate minerals, resulting in additional Ca release during the determination of exchangeable cations [16,17]. Therefore, if residual or incompletely reacted dolomite remained in the soil at sampling, partial carbonate dissolution during NH_4_OAc extraction may also have contributed to the measured exchangeable Ca^2+^ concentrations. However, the Ca and Mg supplied by dolomite at 750 kg ha^−1^ correspond to a maximum of approximately 0.61 cmol(+)·kg^−1^ in the 0–20 cm soil layer, substantially less than the observed N10D–N10CK differences in TEBC (8.28 cmol(+)·kg^−1^) and exchangeable Ca^2+^ (approximately 6.40 cmol(+)·kg^−1^). This quantitative difference indicates that direct amendment-derived Ca and Mg inputs, including their potential release during extraction, cannot fully explain the higher measured exchangeable base-cation concentrations. The observed differences may also reflect changes in soil exchange equilibria and redistribution of pre-existing soil cations associated with acidity neutralization, alongside direct amendment inputs and extraction-related contributions.

Although dolomite and quicklime did not differ significantly in their effects on pHBC, their effects on post-harvest soil pH differed with nitrogen-reduction level. Dolomite and quicklime did not differ significantly under N10, whereas dolomite resulted in higher post-harvest soil pH under N20 and N30. This divergence suggests that their post-harvest effects were not governed solely by acid-neutralizing capacity. Liming materials differ in their neutralizing values and reaction characteristics, and their effects on soil pH are also influenced by soil properties and soil–lime reaction conditions [18,19]. Quicklime is highly reactive, whereas dolomite dissolves more slowly; consequently, differences in reaction kinetics and the persistence of liming effects during the growing season may have contributed to their contrasting post-harvest responses [19].

Interpretation of differences among amendments must also account for the experimental application basis and their contrasting chemical compositions. Because the amendments were applied at an equal mass rather than an equivalent acid-neutralizing dose, their responses should be interpreted within the application conditions evaluated in this study. Quicklime contained a substantially higher proportion of CaO, dolomite supplied both Ca and a higher proportion of MgO, and fused calcium–magnesium phosphate fertilizer additionally supplied phosphorus. Therefore, the contrasting responses among amendments reflect differences in both their acid-neutralizing characteristics and the amounts and forms of base cations and nutrients supplied, rather than an amendment-type effect independent of composition.

### 3.2. Calcium-Based Amendments Reshaped Nutrient Availability and Post-Harvest Inorganic Nitrogen Status Under Nitrogen Reduction

Soil nutrient responses to nitrogen reduction depended on both the nutrient pool considered and amendment application. Under the non-amended condition, SOM was maintained under moderate nitrogen reduction but declined at the highest reduction level, suggesting that excessive nitrogen reduction may compromise post-harvest soil nutrient status. Amendment application modified these responses, consistent with previous field studies showing that moderate nitrogen reduction can maintain soil fertility and crop productivity, whereas greater reductions may adversely affect soil N availability, plant N uptake, or crop yield [20].

Calcium-based amendments further modified nutrient availability, but their effects differed substantially among amendment types. CMP produced a pronounced increase in available phosphorus across nitrogen-reduction levels. Given that CMP itself supplied phosphorus, this response should primarily be attributed to direct phosphorus input rather than interpreted solely as enhanced availability of native soil P [10,21]. In contrast, dolomite was associated with higher SOM and available potassium, particularly under moderate nitrogen reduction. The higher SOM under dolomite may reflect both greater organic C inputs and enhanced stabilization of soil organic matter. Long-term field evidence has shown that dolomitic lime can increase soil organic carbon pools through greater crop-residue C inputs [22] and can alter soil aggregation and carbon-protection mechanisms [23]. These processes provide plausible explanations for the higher SOM observed under dolomite, although their relative contributions cannot be distinguished from the present single-season measurements.

Differences in amendment composition also provide important context for interpreting their contrasting effects on nutrient status. Quicklime contained the highest proportion of CaO, whereas dolomite supplied both Ca and a substantially higher proportion of MgO than CMP. In addition to differences in nutrient inputs, the higher measured CEC under dolomite may reflect differences in soil exchange properties that influence nutrient-cation retention and availability [24,25,26,27,28], potentially contributing to the observed nutrient responses.

Post-harvest nitrogen pools showed a particularly distinct response to dolomite. Dolomite increased alkali-hydrolyzable nitrogen and was associated with higher NH_4_^+^-N and lower NO_3_^−^-N, particularly under moderate nitrogen reduction. The contrasting residual NH_4_^+^-N and NO_3_^−^-N pools may partly reflect differences in their retention and depletion pathways. NH_4_^+^ can be retained on negatively charged exchange sites and, in some soils, fixed within clay minerals [29], whereas NO_3_^−^ is more mobile and may be depleted through plant uptake and other loss pathways during the growing season. Thus, the contrasting NH_4_^+^-N and NO_3_^−^-N concentrations under N20D are better interpreted as differences in the residual pools of these two inorganic N forms rather than as evidence of a direct shift between them.

### 3.3. Balancing Soil Amelioration and Maize Productivity Under Moderate Nitrogen Reduction

The maintenance of maize productivity depended on the extent of nitrogen reduction. Grain yield was maintained when nitrogen reduction increased from 10% to 20%, whereas a further reduction to 30% resulted in a significant yield penalty. This response highlights an important trade-off in acidified croplands: reducing nitrogen input can decrease proton production associated with nitrogen transformations and thereby alleviate acidification pressure [30], but excessive reduction may constrain nutrient supply and ultimately crop productivity [31]. Thus, increasing the degree of nitrogen reduction does not necessarily translate into a better overall soil–crop response. Within the range evaluated here, 20% nitrogen reduction represented a level at which maize productivity could be maintained while reducing nitrogen input.

Calcium-based amendment application further influenced this balance. Dolomite produced the highest marginal mean grain yield across nitrogen-reduction levels, while simultaneously showing favorable responses in several soil properties related to acidity amelioration and nutrient supply. In particular, dolomite was associated with greater pH buffering capacity, higher measured CEC, and higher measured concentrations of exchangeable Ca^2+^ and Mg^2+^, together with favorable responses in nitrogen and potassium availability. Previous studies have similarly shown that liming and dolomite application can alleviate soil acidity, increase exchangeable base-cation availability, improve nutrient status, and support crop productivity in acidic soils [2,14,32,33]. The yield response observed here therefore appears to reflect the combined effects of acidity amelioration and favorable soil nutrient conditions rather than the response to any single soil property.

The multivariate analysis further supports this interpretation while also revealing that soil amelioration and crop productivity represented partly distinct dimensions of the soil–crop response. PC1 was primarily associated with soil acidity and exchangeable Ca^2+^ and Mg^2+^ status, along which dolomite-amended treatments showed the strongest positive shift, particularly under 20% nitrogen reduction. In contrast, maize grain yield was more strongly associated with PC2 and was closely related to SOM. This separation suggests that improvement in acidity-related soil properties alone does not fully explain crop productivity; rather, maintaining yield under reduced nitrogen input requires coordination between soil chemical amelioration and nutrient-supporting conditions. Accordingly, the combined responses observed under 20% nitrogen reduction with dolomite indicate a favorable balance between soil amelioration and maize productivity under the conditions of this experiment. However, because the nitrogen reduction × amendment interaction was not significant for grain yield, this treatment combination should not be interpreted as demonstrating a specific synergistic effect on yield. Moreover, the experiment was conducted at a single site over one growing season; therefore, whether this balance persists under different soils, climatic conditions, and repeated amendment applications requires further multi-year and multi-site evaluation.

## 4. Materials and Methods

### 4.1. Study Area and Soil Characteristics

**Figure 9:**
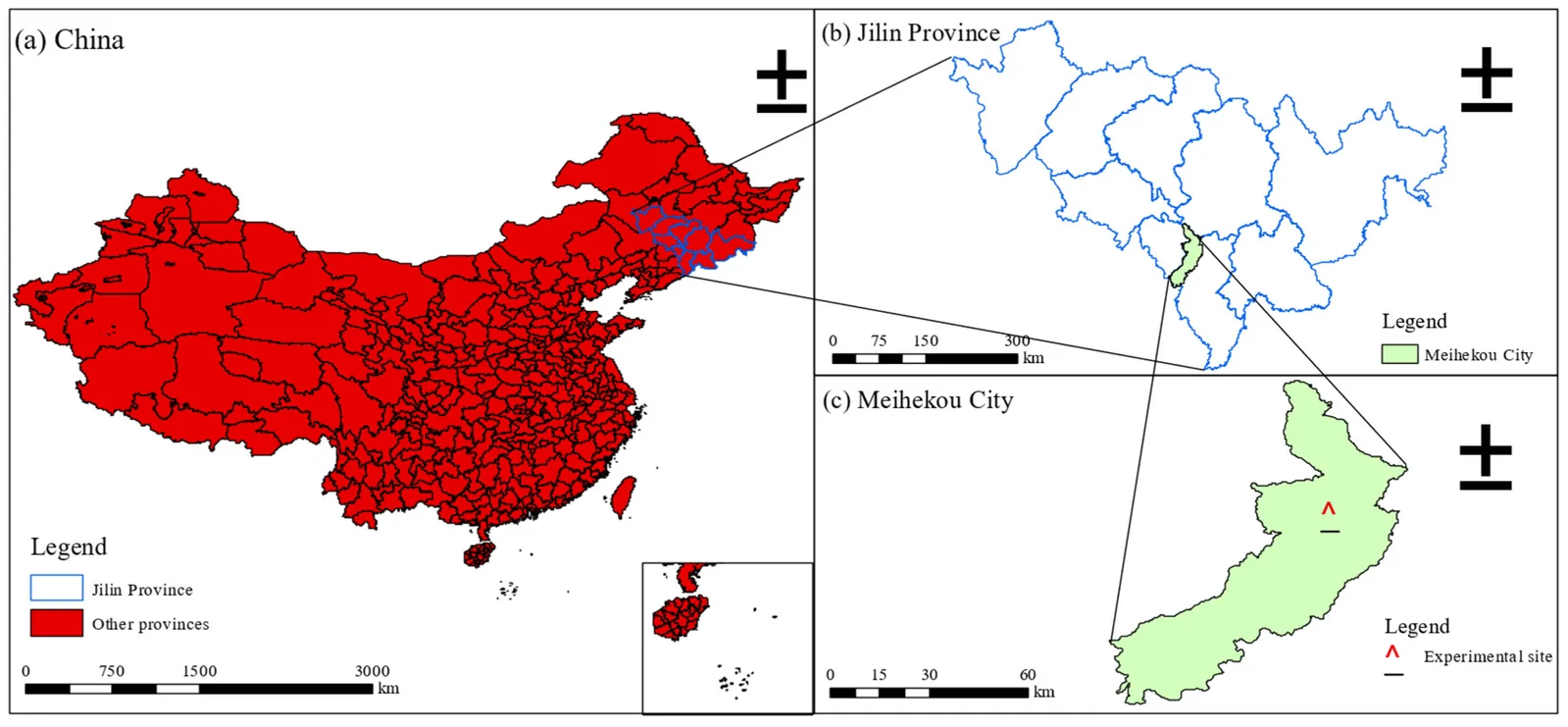
Location of the study area in Meihekou City, Jilin Province, Northeast China.

The field experiment was conducted at an experimental station in Meihekou City, Jilin Province, Northeast China (125°38′ E, 42°32′ N) (Figure 9). Northeast China is one of the major maize-producing regions in China, and maize is the dominant crop cultivated on the land around Meihekou City. The region is characterized by a temperate continental monsoon climate. During the maize growing season from April to October 2025, cumulative precipitation was 759.0 mm and mean air temperature was 16.8 °C, compared with the corresponding 1991–2025 averages of 651.9 mm and 16.1 °C, respectively.

The soil at the experimental site was classified as aquic brown soil according to the Chinese genetic soil classification and approximately corresponds to a Cambisol in the World Reference Base for Soil Resources (WRB) [34]. Before treatment establishment, soil samples were collected from multiple locations within the experimental field at a depth of 0–20 cm and composited to characterize the initial soil physicochemical properties. The selected physicochemical properties of the soil were as follows: pH, 5.09; bulk density, 1.33 g·cm^−3^; cation exchange capacity (CEC), 15.39 cmol(+)·kg^−1^; total exchangeable base cations, 7.98 cmol(+)·kg^−1^; exchangeable acidity, 0.68 cmol(+)·kg^−1^; soil organic matter, 27.20 g·kg^−1^; total nitrogen, 1.44 g·kg^−1^; alkali-hydrolyzable nitrogen, 122.68 mg·kg^−1^; total phosphorus, 0.59 g·kg^−1^; and total potassium, 15.38 g·kg^−1^.

### 4.2. Experimental Design and Treatments

A factorial field experiment was conducted from April to October 2025 during the maize growing season using a randomized complete block design with three replicates to evaluate the effects of nitrogen reduction and calcium-based amendments on soil acidification and maize productivity. The conventional nitrogen application level in the region (240 kg·N·ha^−1^) was used as the reference level. Three nitrogen-reduction levels were established, corresponding to reductions of 10%, 20%, and 30%, with nitrogen application levels of 216, 192, and 168 kg·N·ha^−1^, respectively. These treatments were designated as N10, N20, and N30.

Three calcium-based soil amendments, namely dolomite, quicklime, and CMP, were applied at the same rate of 750 kg·ha^−1^. A non-amended control was included. The amendment treatments were coded as D, Q, C, and CK, representing dolomite, quicklime, CMP, and no amendment, respectively. The basic chemical properties of the amendments were as follows: dolomite (CaO ≥ 30.4%, MgO ≥ 21.9%, pH 11.38; Shandong Runjie New Materials Technology Co., Ltd., Jinan, China), quicklime (CaO ≥ 99.6%, pH 13.89; Yiyang Heshan District Huangmao Trading Co., Ltd., Yiyang, China), and CMP (P_2_O_5_ ≥ 12%, CaO ≥ 20%, MgO ≥ 3%, SiO_2_ ≥ 10%, pH 11.26; Jingmen Gaoyuan Phosphate Fertilizer Co., Ltd., Zhongxiang, China). At the applied rate, CMP supplied approximately 90 kg P_2_O_5_ ha^−1^ in addition to the basal phosphorus fertilizer applied uniformly to all treatments. The amendment rate corresponded to approximately 0.028% (*w*/*w*) in the 0–20 cm soil layer based on soil bulk density.

The experiment consisted of twelve treatment combinations generated by the factorial arrangement of three nitrogen-reduction levels and four amendment regimes (Table 1). Treatment codes were assigned according to nitrogen-reduction level and amendment type (e.g., N20D represents 20% nitrogen reduction combined with dolomite application). Each experimental plot measured 8 m × 6 m (48 m^2^), with a 1 m buffer zone maintained between adjacent plots to minimize potential interference among treatments.

### 4.3. Field Management

Maize (*Zea mays* L., cultivar GD 2003) was planted at a planting density of 52,500 plants·ha^−1^. Phosphorus fertilizer (diammonium phosphate, Hubei Yihua International Trading Co., Ltd., Yichang, China) and potassium fertilizer (potassium chloride, Liaoning Migao Chemical Co., Ltd., Yingkou, China) were uniformly applied to all plots at levels of 90 kg P_2_O_5_ ha^−1^ and 90 kg K_2_O ha^−1^, respectively. Nitrogen fertilizer (urea, Inner Mongolia Ordos United Chemical Co., Ltd., Ordos, China) was applied according to the predefined treatment levels described in Section 4.2 and was split into two applications, with one-third applied as basal fertilizer and two-thirds applied as topdressing during the growing season.

Calcium-based amendments were incorporated into the soil before sowing according to the treatment design. All plots were managed consistently throughout the growing season. The field experiment was rainfed, with crop water supply relying on natural precipitation. Weeds were manually removed when necessary to minimize competition with maize. No pesticide application was required during the growing season because no major pest or disease outbreaks occurred.

### 4.4. Soil Sampling and Soil Property Measurements

Soil samples were collected after maize harvest in October 2025 from the 0–20 cm soil layer. In each plot, five soil cores were randomly collected using a stainless-steel auger and thoroughly mixed to obtain one composite sample. Visible roots and plant residues were removed before sample processing. The soil samples were air-dried, gently ground, and passed through a 2 mm sieve for subsequent physicochemical analyses.

#### 4.4.1. Soil Acidification Indicators

Soil pH was measured in a soil–water suspension at a ratio of 1:2.5 (*w*/*v*) using a calibrated pH meter (PHS-3C, Shanghai INESA Scientific Instrument Co., Ltd., Shanghai, China) [35]. For both the initial soil composite and post-harvest soil samples, cation exchange capacity (CEC) was determined using the ammonium acetate (NH_4_OAc) saturation–distillation titration method (1 mol L^−1^ NH_4_OAc, pH 7.0), whereas exchangeable acidity was determined by extraction with 1 mol L^−1^ KCl, followed by titration with standardized NaOH [35]. Soil pH buffering capacity (pHBC) was determined using the acid titration curve method. Briefly, 10 g of air-dried soil was treated with sulfuric acid solution at an H^+^ equivalent concentration of 0.2 mol L^−1^ (corresponding to 0.1 mol L^−1^ H_2_SO_4_) at 11 acid-addition levels (0–100 mmol H^+^ kg^−1^ soil, with 10 mmol H^+^ kg^−1^ intervals). Deionized water was added to maintain a soil-to-solution ratio of 1:2.5. The suspensions were equilibrated at 25 °C by shaking for 6 h and standing for 2 h, with this cycle repeated three times, followed by shaking for 24 h. Equilibrium pH was then measured. The complete 11-point titration curves were used to characterize soil pH responses across the full acid-addition gradient. For pHBC estimation, linear regression was restricted to the low-acid-addition range (≤20 mmol H^+^ kg^−1^ soil) and fitted using the following equation [13,36]:

(1) y=ax+b

where *y* represents soil pH, *x* represents the amount of added acid expressed as H^+^ equivalents (mmol H^+^ kg^−1^), and *a* and *b* represent the slope and intercept of the fitted regression, respectively. Soil pH buffering capacity was calculated as the reciprocal of the absolute value of the slope:

(2) $$\mathrm{pHBC}=\frac{1}{\left|a\right|}$$

where pHBC is expressed as mmol H^+^ kg^−1^ pH^−1^. The slope, its standard error, and the coefficient of determination (*R*^2^) were obtained separately for each experimental replicate from the low-acid-range linear regression.

#### 4.4.2. Exchangeable Base Cations

Exchangeable base cations were determined according to the soil agrochemical analysis methods described by Lu [35]. Exchangeable Ca^2+^, Mg^2+^, K^+^, and Na^+^ were extracted using 1 mol L^−1^ ammonium acetate solution (pH 7.0). The concentrations of K^+^ and Na^+^ were determined using flame photometry (FP6410, INESA Analytical Instrument Co., Ltd., Shanghai, China), whereas Ca^2+^ and Mg^2+^ were measured using atomic absorption spectrophotometry (Z-2000, Hitachi High-Tech Corporation, Tokyo, Japan). Total exchangeable base cations (TEBC) were calculated as the sum of exchangeable Ca^2+^, Mg^2+^, K^+^, and Na^+^.

#### 4.4.3. Soil Nutrient Availability

Soil ammonium nitrogen (NH_4_^+^-N) and nitrate nitrogen (NO_3_^−^-N) were extracted using 0.01 mol L^−1^ CaCl_2_ solution and analyzed using an AA3 continuous flow analyzer (Seal Analytical, Wrexham, UK) [37].

Soil organic carbon (SOC) was determined using a UNICUBE elemental analyzer (Elementar, Langenselbold, Germany), and soil organic matter (SOM) was calculated using the conventional conversion factor of 1.724 [35,38] as follows:

(3) SOM=SOC×1.724

Alkali-hydrolyzable nitrogen was determined using the alkaline diffusion method [35]. Available phosphorus was extracted with 0.5 mol L^−1^ NaHCO_3_ and determined using the molybdenum–antimony–ascorbic acid colorimetric method [37,39]. Available potassium was extracted with 1 mol L^−1^ NH_4_OAc and measured using flame photometry (FP6410, Inesa, China) [35].

### 4.5. Plant Sampling and Yield Determination

At the physiological maturity stage of maize (110–130 days post-planting, when seed kernels reach their maximum dry weight and a protective black layer forms at their base), grain yield was determined for each plot. Five representative ears were randomly collected from each plot and manually threshed to determine grain moisture content. Maize plants within the harvest area of each plot were then harvested and threshed, and the total grain weight was recorded. Grain yield was adjusted to a 14% moisture content and expressed as t·ha^−1^ [40].

### 4.6. Statistical Analysis

Treatment effects were analyzed using two-factor analysis of variance (ANOVA) for a randomized complete block design, with nitrogen-reduction level, calcium-based amendment, and their interaction included as fixed effects and block included in the model. When the interaction was significant, simple-effects analyses were conducted by comparing amendment treatments within each nitrogen-reduction level and nitrogen-reduction levels within each amendment treatment. Pairwise comparisons were adjusted using Tukey’s honestly significant difference (HSD) procedure at *p* < 0.05.

Principal component analysis (PCA) was performed using variables representing soil acidity and buffering, exchangeable base-cation status, nutrient availability, and maize productivity to characterize their multivariate responses to nitrogen reduction and calcium-based amendment treatments. All variables were Z-score standardized before PCA, which was conducted based on the correlation matrix [41,42]. Principal components with eigenvalues > 1 were retained according to the Kaiser criterion [43], and variable loadings were used to interpret the retained components [44]. Pearson correlation analysis was performed to assess relationships among the variables included in the PCA, with statistical significance evaluated at *p* < 0.05.

All statistical analyses were performed using SPSS version 19.0 (IBM Corp., Armonk, NY, USA), and figures were generated using Origin 2024 (OriginLab Corporation, Northampton, MA, USA).

## 5. Conclusions

This study showed that nitrogen reduction and calcium-based amendment application affected different components of the soil–crop system, with responses varying according to nitrogen-reduction intensity and amendment type. Increasing nitrogen reduction alleviated soil acidification pressure under non-amended conditions, but greater reduction did not produce a corresponding improvement in soil buffering capacity, and a 30% reduction resulted in a significant decline in maize yield. The amendments differed in their effects on soil acidity and nutrient status: dolomite was associated with higher pH buffering capacity, higher post-harvest CEC values, higher measured exchangeable Ca^2+^ and Mg^2+^ concentrations, and favorable responses in soil nutrient availability, whereas the higher available phosphorus under fused calcium–magnesium phosphate fertilizer was largely attributable to the additional phosphorus supplied by the amendment. Within the treatments evaluated, combining 20% nitrogen reduction with dolomite was associated with a favorable balance among soil acidity amelioration, soil buffering, nutrient status, and maize productivity. These findings highlight the importance of coordinating nitrogen-reduction intensity with amendment-specific effects rather than considering nitrogen reduction or soil acidity amelioration alone. Because the experiment was conducted at a single site over one growing season, the observed responses represent short-term effects under the soil and climatic conditions evaluated, and their consistency should be further assessed through multi-year and multi-site field experiments.

## Figures and Tables

**Table 1:** Experimental treatments of nitrogen reduction combined with different calcium-based amendments.

Treatment Code	Nitrogen Reduction (%)	Nitrogen Level (kg·ha^−1^)	Amendment Type	Amendment (kg·ha^−1^)
N10CK	10	216	None	0
N20CK	20	192	None	0
N30CK	30	168	None	0
N10D	10	216	Dolomite	750
N20D	20	192	Dolomite	750
N30D	30	168	Dolomite	750
N10Q	10	216	Quicklime	750
N20Q	20	192	Quicklime	750
N30Q	30	168	Quicklime	750
N10C	10	216	Fused calcium–magnesium phosphate fertilizer	750
N20C	20	192	Fused calcium–magnesium phosphate fertilizer	750
N30C	30	168	Fused calcium–magnesium phosphate fertilizer	750

## Data Availability

The data presented in this study are available in the article and Supplementary Materials. Additional data are available from the corresponding author upon reasonable request.

## Supplementary Materials

The following supporting information can be downloaded at: https://www.mdpi.com/article/10.3390/plants15203116/s1. Table S1: Plot-level low-acid-range regression parameters and soil pH buffering capacity (pHBC) under different nitrogen-reduction and calcium-based amendment treatments. Table S2: Plot-level exchangeable Ca^2+^, Mg^2+^, K^+^, and Na^+^ concentrations and total exchangeable base cations (TEBC) under different nitrogen-reduction and calcium-based amendment treatments (cmol(+) kg^−1^). Table S3: Post-harvest total exchangeable base cations (TEBC), cation exchange capacity (CEC), exchangeable acidity, and base saturation under different nitrogen-reduction and calcium-based amendment treatments. Table S4: Eigenvalues and variance explained by the retained principal components in the principal component analysis of soil properties and maize grain yield. Table S5: Loadings of soil acidity indicators, nutrient availability, and maize productivity variables on the retained principal components (see Figure 8 in the main manuscript). Table S6: Pearson correlation matrix among soil properties and maize grain yield included in the principal component analysis. Figure S1: Full acid-titration responses of soils under nitrogen reduction and calcium-based amendment treatments. Symbols represent individual observations across the full acid-addition range. N10, N20, and N30 denote 10%, 20%, and 30% nitrogen reduction, respectively; CK, D, Q, and C denote no amendment, dolomite, quicklime, and fused calcium–magnesium phosphate fertilizer, respectively. Figure S2: Heatmap of normalized exchangeable base-cation concentrations under different nitrogen-reduction and calcium-based amendment treatments. Mean concentrations of exchangeable Ca^2+^, Mg^2+^, K^+^, and Na^+^ for each treatment combination were normalized separately for each ion for visualization. N10, N20, and N30 represent 10%, 20%, and 30% reductions in nitrogen application, respectively; CK, D, Q, and C represent no amendment, dolomite, quicklime, and fused calcium–magnesium phosphate fertilizer, respectively.

## References

[1] Shen J., Luo Y., Tao Q., White P.J., Sun G., Li M., Luo J., He Y., Li B., Li Q. (2022). The exacerbation of soil acidification correlates with structural and functional succession of the soil microbiome upon agricultural intensification. Sci. Total Environ..

[2] Zhang S., Zhu Q., de Vries W., Ros G.H., Chen X., Muneer M.A., Zhang F., Wu L. (2023). Effects of soil amendments on soil acidity and crop yields in acidic soils: A world-wide meta-analysis. J. Environ. Manag..

[3] Raza S., Miao N., Wang P., Ju X., Chen Z., Zhou J., Kuzyakov Y. (2020). Dramatic loss of inorganic carbon by nitrogen-induced soil acidification in Chinese croplands. Glob. Change Biol..

[4] Xu Y., Yu Z., Li Y., Jin J., Zhang X., Wang G., Liu X. (2024). Removal of crop ion components in relation to Mollisol acidification under long-term management. Agronomy.

[5] Kochian L.V., Piñeros M.A., Liu J., Magalhaes J.V. (2015). Plant adaptation to acid soils: The molecular basis for crop aluminum resistance. Annu. Rev. Plant Biol..

[6] Dong Y., Yang J.L., Zhao X.R., Yang S.H., Mulder J., Dörsch P., Zhang G.L. (2022). Seasonal dynamics of soil pH and N transformation as affected by N fertilization in subtropical China: An in situ ^15^N-labeling study. Sci. Total Environ..

[7] Pramanick B., Choudhary S., Kumar M., Singh S.K., Jha R.K., Singh S.K., Salmen S.H., Ansari M.J., Hossain A. (2024). Can site-specific nutrient management improve the productivity and resource use efficiency of climate-resilient finger millet in calcareous soils in India?. Heliyon.

[8] Zhang Y., Ye C., Su Y., Peng W., Lu R., Liu Y., Huang H., He X., Yang M., Zhu S. (2022). Soil acidification caused by excessive application of nitrogen fertilizer aggravates soil-borne diseases: Evidence from literature review and field trials. Agric. Ecosyst. Environ..

[9] Shaaban M., Wang X., Song P., Hu R., Wu Y. (2024). Impact of dolomite liming on ammonia-oxidizing microbial populations and soil biochemistry in acidic rice paddy soils. Agronomy.

[10] Chen C., Xiang Y., Jiao X., Gong H. (2024). Enhancing maize phosphorus uptake with optimal blends of high- and low-concentration phosphorus fertilizers. Front. Plant Sci..

[11] Zhao W., Hu W., Zhang F., Shi Y., Wang Y., Zhang X., Feng T., Hong Z., Jiang J., Xu R. (2024). Exchangeable acidity characteristics of farmland black soil in Northeast China. Geoderma Reg..

[12] He X., Nkoh J.N., Shi R.Y., Xu R.K. (2022). Application of chitosan- and alginate-modified biochars in promoting the resistance to paddy soil acidification and immobilization of soil cadmium. Environ. Pollut..

[13] Wei H., Yang J., Liu Z., Zhang J. (2022). Data integration analysis indicates that soil texture and pH greatly influence the acid-buffering capacity of global surface soils. Sustainability.

[14] Qaswar M., Li D., Huang J., Han T., Ahmed W., Abbas M., Zhang L., Du J., Khan Z.H., Ullah S. (2020). Interaction of liming and long-term fertilization increased crop yield and phosphorus use efficiency (PUE) through mediating exchangeable cations in acidic soil under wheat–maize cropping system. Sci. Rep..

[15] Wada S.I., Furumura S. (1994). Solubility of CaCO_3_ in 1 mol L^−1^ ammonium acetate for extracting exchangeable bases. Soil Sci. Plant Nutr..

[16] Lalitha M., Parvathy S., Koyal A., Hegde R., Ramamurthy V. (2026). Impact of calcium carbonate solubility on estimating soil exchangeable cations in calcareous soils using 1N ammonium acetate method. Commun. Soil Sci. Plant Anal..

[17] Edmeades D.C. (1982). Effects of lime on effective cation exchange capacity and exchangeable cations on a range of New Zealand soils. N. Z. J. Agric. Res..

[18] Han D., Zeng S., Zhang X., Li J., Ma Y. (2023). Integrating soil pH, clay, and neutralizing value of lime into a new lime requirement model for acidic soils in China. Agronomy.

[19] Wenyika P., Enesi R.O., Gorim L.Y., Dyck M. (2025). Effects of liming on soil physical and chemical properties in Europe and North America: A review. Agrosyst. Geosci. Environ..

[20] Wang P., Yu A., Jiang K., Pang X., Huo J., Wang Y., Shang Y., Hou S. (2026). Green manure incorporation with 20% nitrogen reduction enhances soil organic carbon storage by improving aggregate stability and functional group composition. Agric. Ecosyst. Environ..

[21] Alemu E., Selassie Y.G., Yitaferu B. (2022). Effect of lime on selected soil chemical properties, maize (*Zea mays* L.) yield and determination of rate and method of its application in Northwestern Ethiopia. Heliyon.

[22] Briedis C., de Moraes Sá J.C., Caires E.F., de Fátima Navarro J., Inagaki T.M., Boer A., de Oliveira Ferreira A., Neto C.Q., Canalli L.B., dos Santos J.B. (2012). Changes in organic matter pools and increases in carbon sequestration in response to surface liming in an Oxisol under long-term no-till. Soil Sci. Soc. Am. J..

[23] Briedis C., de Moraes Sá J.C., Caires E.F., de Fátima Navarro J., Inagaki T.M., Boer A., Neto C.Q., de Oliveira Ferreira A., Canalli L.B., dos Santos J.B. (2012). Soil organic matter pools and carbon-protection mechanisms in aggregate classes influenced by surface liming in a no-till system. Geoderma.

[24] Yang Z., Zhou W., Sun B., Rengel Z., Feng G., Zhang L. (2023). Combined application of calcium-magnesium phosphate fertilizer with soluble phosphorus improves maize growth in a calcareous soil. J. Soil Sci. Plant Nutr..

[25] Wu H., Hu J., Shaaban M., Xu P., Zhao J., Hu R. (2021). The effect of dolomite amendment on soil organic carbon mineralization is determined by the dolomite size. Ecol. Process..

[26] Suriyagoda L.D.B., Sirisena D.N., Somaweera K.A.T.N., Dissanayake A., De Costa W.A.J.M., Lambers H. (2017). Incorporation of dolomite reduces iron toxicity, enhances growth and yield, and improves phosphorus and potassium nutrition in lowland rice (*Oryza sativa* L.). Plant Soil.

[27] Han T., Cai A., Liu K., Huang J., Wang B., Li D., Zhang H. (2019). The links between potassium availability and soil exchangeable calcium, magnesium, and aluminum are mediated by lime in acidic soil. J. Soils Sediments.

[28] Hardie A.G., de Bod R., Raath P.J. (2026). Comparison of movement of alkalinity from surface applied biochar, wood ash and dolomitic limestone in acid sandy loam soil. J. Soil Sci. Plant Nutr..

[29] Nieder R., Benbi D.K., Scherer H.W. (2011). Fixation and defixation of ammonium in soils: A review. Biol. Fertil. Soils.

[30] Guo J.H., Liu X.J., Zhang Y., Shen J.L., Han W.X., Zhang W.F., Christie P., Goulding K.W., Vitousek P.M., Zhang F.S. (2010). Significant acidification in major Chinese croplands. Science.

[31] Kang J., Chu Y., Ma G., Zhang Y., Zhang X., Wang M., Lu H., Wang L., Kang G., Ma D. (2023). Physiological mechanisms underlying reduced photosynthesis in wheat leaves grown in the field under conditions of nitrogen and water deficiency. Crop J..

[32] Bossolani J.W., Crusciol C.A.C., Momesso L., Portugal J.R., Moretti L.G., Garcia A., da Fonseca M.C., Rodrigues V.A., Calonego J.C., dos Reis A.R. (2022). Surface liming triggers improvements in subsoil fertility and root distribution to boost maize crop physiology, yield and revenue. Plant Soil.

[33] Kasno A., Nurida N., Siregar A.F., Samsun A., Widowati L.R., Husnain H. (2023). Enhancing chemical properties and maize yield through dolomite application on rock phosphate-amended oxisol. E3S Web Conf..

[34] Shi X.Z., Yu D.S., Warner E.D., Pan X.Z., Petersen G.W., Gong Z.G., Weindorf D.C. (2004). Soil database of 1:1,000,000 digital soil survey and reference system of the Chinese genetic soil classification system. Soil Surv. Horiz..

[35] Lu R.K. (2000). Methods of Soil Agricultural Chemical Analysis.

[36] Zhu R., Huang J., Liu J., Xiao M., Li A. (2026). Tropical N-poor soils have strong acidity buffering capacity for short-term increased and decreased nitrogen deposition. Plant Soil.

[37] Zhu L., Liang A., Wang R., Shi Y., Li J., Wang R., Wang M., Guo S. (2023). Harnessing nitrate over ammonium to sustain soil health during monocropping. Front. Plant Sci..

[38] Pribyl D.W. (2010). A critical review of the conventional SOC to SOM conversion factor. Geoderma.

[39] Zhou L., Zhu L., Zhang F. (2026). Phytase and phosphate-solubilizing bacteria addition improves P availability and uptake by maize in low-phosphorus red soil. PLoS ONE.

[40] Shen D., Wang K., Zhou L., Fang L., Wang Z., Fu J., Zhang T., Liang Z., Xie R., Ming B. (2024). Increasing Planting Density and Optimizing Irrigation to Improve Maize Yield and Water-Use Efficiency in Northeast China. Agronomy.

[41] Andrews S.S., Karlen D.L., Mitchell J.P. (2002). A comparison of soil quality indexing methods for vegetable production systems in Northern California. Agric. Ecosyst. Environ..

[42] Faloye O.T., Ajayi A.E., Kamchoom V., Akintola O.A., Oguntunde P.G. (2024). Evaluating impacts of biochar and inorganic fertilizer applications on soil quality and maize yield using principal component analysis. Agronomy.

[43] Kaiser H.F. (1960). The application of electronic computers to factor analysis. Educ. Psychol. Meas..

[44] Jolliffe I.T., Cadima J. (2016). Principal Component Analysis: A Review and Recent Developments. Philos. Trans. R. Soc. A Math. Phys. Eng. Sci..

