# Supplementary material for: Nitrogen Reduction Combined with Calcium-Based Amendments Regulates Soil Acidification Buffering and Maize Productivity in Acidified Croplands of Northeast China
Source: Plants (Basel). 2026 Oct 12;15(20):3116. doi: 10.3390/plants15203116 (PMC13709314; doi:10.3390/plants15203116)
Supplement: Supplementary file 1 [file plants-15-03116-s001.zip › plants-4573266-supplementary.pdf]

Nitrogen reduction combined with calcium-based amendments regulates soil acidification buffering and maize productivity in acidified croplands of Northeast China

Yiting Liu [1], Letian Jin [1], Na Yuan [1], Mengmeng Bao [1], Fengzhuo Geng [1], Dongran Liu [1], Jiachen Tong [1], Iseult Lynch [2] and Xiuli Dang [1*]

[1] National Engineering Research Center for Efficient Utilization of Soil and Fertilizer Resources, Key La-boratory of Arable Land Conservation in Northeast China, Ministry of Agriculture and Rural Affairs, College of Land and Environment, Shenyang Agricultural University, Shenyang 110866, China; 2024220437@stu.syau.edu.cn (Y.L.); jlt2000@syau.edu.cn (L.J.); yn0121@stu.syau.edu.cn (N.Y.); bmm121@stu.syau.edu.cn (M.B.); 2024220452@stu.syau.edu.cn (F.G.); imnot1iudong@syau.edu.cn (D.L.); 2025240676@stu.syau.edu.cn (J.T.).

[2] School of Geography, Earth and Environmental Sciences, University of Birmingham, Edgbaston, Birmingham, B15 2TT, UK; i.lynch@bham.ac.uk (I.L.)

* Correspondence: dxl@syau.edu.cn；Tel: +86-136-0492-2085

The number of pages: 10

The number of tables: 6

The number of figures: 2

List of Supporting Information

Tables:

Table S1. Plot-level low-acid-range regression parameters and soil pH buffering capacity (pHBC) under different nitrogen-reduction and calcium-based amendment treatments.

Table S2. Plot-level exchangeable Ca^{2+}, Mg^{2+}, K^{+}, and Na^{+} concentrations and total exchangeable base cations (TEBC) under different nitrogen reduction and calcium-based amendment treatments (cmol(+)·kg^{-1}).

Table S3. Post-harvest total exchangeable base cations (TEBC), cation exchange capacity (CEC), exchangeable acidity, and base saturation under different nitrogen-reduction and calcium-based amendment treatments.

Table S4. Eigenvalues and variance explained by the retained principal components in the principal component analysis of soil properties and maize grain yield.

Table S5. Loadings of soil acidity indicators, nutrient availability, and maize productivity variables on the retained principal components (see Figure 8 in the main manuscript).

Table S6. Pearson correlation matrix among soil properties and maize grain yield included in the principal component analysis.

Figure:

Figure S1. Full acid-titration responses of soils under nitrogen reduction and calcium-based amendment treatments. Symbols represent individual observations across the full acid-addition range. N10, N20, and N30 denote 10%, 20%, and 30% nitrogen reduction, respectively; CK, D, Q, and C denote no amendment, dolomite, quicklime, and fused calcium–magnesium phosphate fertilizer, respectively.

Figure S2. Heatmap of normalized exchangeable base-cation concentrations under different nitrogen reduction and calcium-based amendment treatments. Mean

concentrations of exchangeable Ca^{2+}, Mg^{2+}, K^{+}, and Na^{+} for each treatment combination were normalized separately for each ion using Normalized value. N10, N20, and N30 represent 10%, 20%, and 30% reductions in nitrogen application, respectively; CK, D, Q, and C represent no amendment, dolomite, quicklime, and fused calcium–magnesium phosphate fertilizer, respectively.

Table S1. Plot-level low-acid-range regression parameters and soil pH buffering capacity (pHBC) under different nitrogen-reduction and calcium-based amendment treatments.

Treatment	Replicate	Slope (*a*)	Standard Error (SE)	R^2	pHBC
N10CK	1	-0.08750	0.00606	0.99522	11.43
N10CK	2	-0.09700	0.00808	0.99310	10.31
N10CK	3	-0.09700	0.00924	0.99101	10.31
N20CK	1	-0.08500	0.00808	0.99104	11.76
N20CK	2	-0.08450	0.00087	0.99989	11.83
N20CK	3	-0.08400	0.00693	0.99324	11.90
N30CK	1	-0.06700	0.02021	0.91662	14.93
N30CK	2	-0.09550	0.01530	0.97498	10.47
N30CK	3	-0.09900	0.01790	0.96835	10.10
N10D	1	-0.08850	0.01415	0.97509	11.30
N10D	2	-0.06600	0.01674	0.93954	15.15
N10D	3	-0.08200	0.00693	0.99291	12.20
N20D	1	-0.07350	0.00664	0.99191	13.61
N20D	2	-0.08600	0.00462	0.99712	11.63
N20D	3	-0.07750	0.00491	0.99601	12.90
N30D	1	-0.07350	0.00375	0.99740	13.61
N30D	2	-0.07400	0.00289	0.99848	13.51
N30D	3	-0.06800	0.00404	0.99648	14.71
N10Q	1	-0.08950	0.00144	0.99974	11.17
N10Q	2	-0.07150	0.00202	0.99920	13.99
N10Q	3	-0.07500	0.00346	0.99787	13.33
N20Q	1	-0.08350	0.00953	0.98715	11.98
N20Q	2	-0.07150	0.00202	0.99920	13.99
N20Q	3	-0.06850	0.00087	0.99984	14.60
N30Q	1	-0.07200	0.00173	0.99942	13.89
N30Q	2	-0.07300	0.00404	0.99694	13.70
N30Q	3	-0.06900	0.00924	0.98239	14.49
N10C	1	-0.08950	0.00318	0.99874	11.17
N10C	2	-0.07700	0.00000	1.00000	12.99
N10C	3	-0.07150	0.00202	0.99920	13.99
N20C	1	-0.09150	0.01010	0.98795	10.93
N20C	2	-0.08450	0.00722	0.99276	11.83
N20C	3	-0.07950	0.00606	0.99422	12.58
N30C	1	-0.08100	0.00058	0.99995	12.35
N30C	2	-0.08350	0.01357	0.97428	11.98
N30C	3	-0.07950	0.01126	0.98034	12.58

Note: N10, N20, and N30 represent 10%, 20%, and 30% reductions in nitrogen application, respectively; CK, D, Q, and C represent no amendment, dolomite, quicklime, and fused calcium–magnesium phosphate fertilizer, respectively.

Table S2. Plot-level exchangeable Ca^{2+}, Mg^{2+}, K^{+}, and Na^{+} concentrations and total exchangeable base cations (TEBC) under different nitrogen reduction and calcium-based amendment treatments (cmol(+)·kg^{-1}).

Treatment	Plot	Exchangeable Ca^{2+}	Exchangeable Mg^{2+}	Exchangeable K^{+}	Exchangeable Na^{+}	TEBC
N10CK	1	4.33	0.95	0.14	0.22	5.64
N10CK	2	4.75	0.98	0.07	0.17	5.97
N10CK	3	4.57	1.05	0.10	0.18	5.90
N20CK	1	6.25	1.78	0.22	0.21	8.46
N20CK	2	6.44	1.82	0.28	0.22	8.76
N20CK	3	6.52	1.88	0.25	0.20	8.85
N30CK	1	7.39	1.86	0.23	0.25	9.73
N30CK	2	7.00	1.82	0.20	0.26	9.28
N30CK	3	7.79	1.92	0.21	0.26	10.18
N10D	1	10.62	2.70	0.30	0.13	13.75
N10D	2	11.21	2.76	0.30	0.12	14.39
N10D	3	11.01	2.80	0.27	0.13	14.21
N20D	1	12.01	3.17	0.30	0.15	15.63
N20D	2	11.95	3.21	0.37	0.14	15.67
N20D	3	12.30	3.28	0.33	0.14	16.05
N30D	1	11.81	3.07	0.28	0.12	15.28
N30D	2	10.81	3.15	0.25	0.17	14.38
N30D	3	12.81	3.25	0.24	0.14	16.44
N10Q	1	8.30	1.87	0.10	0.14	10.41
N10Q	2	8.60	1.99	0.15	0.16	10.90
N10Q	3	8.47	1.91	0.13	0.19	10.70
N20Q	1	9.37	1.96	0.16	0.14	11.63
N20Q	2	10.27	2.07	0.20	0.20	12.74
N20Q	3	9.07	2.02	0.24	0.17	11.50
N30Q	1	9.87	2.24	0.20	0.17	12.48
N30Q	2	9.27	2.28	0.24	0.15	11.94
N30Q	3	11.07	2.34	0.29	0.19	13.89
N10C	1	7.53	2.27	0.12	0.18	10.10
N10C	2	8.20	2.38	0.13	0.14	10.85
N10C	3	7.46	2.35	0.13	0.16	10.10
N20C	1	9.08	2.48	0.24	0.20	12.00
N20C	2	8.92	2.58	0.20	0.12	11.82
N20C	3	9.68	2.54	0.24	0.16	12.62
N30C	1	10.10	2.54	0.13	0.13	12.90
N30C	2	10.36	2.55	0.13	0.17	13.21
N30C	3	10.20	2.65	0.14	0.23	13.22

Note: N10, N20, and N30 represent 10%, 20%, and 30% reductions in nitrogen application, respectively; CK, D, Q, and C represent no amendment, dolomite, quicklime, and fused calcium–magnesium phosphate fertilizer, respectively.

Table S3. Post-harvest total exchangeable base cations (TEBC), cation exchange capacity (CEC), exchangeable acidity, and base saturation under different nitrogen-reduction and calcium-based amendment treatments.

Treatment	TEBC ($cmol(+)\cdot kg^{-1}$)	CEC ($cmol(+)\cdot kg^{-1}$)	Exchangeable acidity ($cmol(+)\cdot kg^{-1}$)	Base saturation (%)
N10CK	5.84 ± 0.10	18.35 ± 0.58	3.50 ± 0.16	31.85 ± 0.78
N20CK	8.69 ± 0.12	16.07 ± 0.56	3.01 ± 0.15	54.20 ± 1.68
N30CK	9.73 ± 0.26	19.80 ± 1.02	2.96 ± 0.19	49.28 ± 1.35
N10D	14.12 ± 0.19	28.60 ± 0.64	2.05 ± 0.17	49.37 ± 0.61
N20D	15.78 ± 0.13	33.19 ± 0.94	1.51 ± 0.19	47.61 ± 1.20
N30D	15.37 ± 0.60	32.85 ± 1.77	1.76 ± 0.10	46.97 ± 2.37
N10Q	10.67 ± 0.14	22.44 ± 1.07	1.80 ± 0.16	47.81 ± 2.79
N20Q	11.96 ± 0.39	20.92 ± 0.48	1.40 ± 0.19	57.12 ± 0.57
N30Q	12.77 ± 0.58	18.09 ± 0.65	1.32 ± 0.16	70.74 ± 3.78
N10C	10.35 ± 0.25	18.69 ± 0.76	2.88 ± 0.25	55.62 ± 3.22
N20C	12.15 ± 0.24	17.13 ± 0.74	2.70 ± 0.17	71.06 ± 1.99
N30C	13.11 ± 0.11	16.55 ± 0.78	2.63 ± 0.16	79.50 ± 3.18

Note: Values are means ± SE (n = 3 independent field plots). Base saturation was calculated for each plot as TEBC/CEC × 100, and the treatment mean ± SE is reported. N10, N20, and N30 represent 10%, 20%, and 30% reductions in nitrogen application, respectively; CK, D, Q, and C represent no amendment, dolomite, quicklime, and fused calcium–magnesium phosphate fertilizer, respectively.

Table S4. Eigenvalues and variance explained by the retained principal components in the principal component analysis of soil properties and maize grain yield.

Principal component	Eigenvalue	Variance explained (%)	Cumulative variance explained (%)
PC1	6.602	50.78	50.78
PC2	1.958	15.06	65.84
PC3	1.236	9.51	75.35

Note: Principal components with eigenvalues > 1 were retained.

Table S5. Loadings of soil acidity indicators, nutrient availability, and maize productivity variables on the retained principal components (see Figure 8 in the main manuscript).

Variable	PC1	PC2	PC3
Soil pH	0.338	−0.230	0.042
pHBC	0.208	−0.216	0.184
Exchangeable Ca^{2+}	0.341	−0.261	0.018
Exchangeable Mg^{2+}	0.344	−0.196	−0.024

Variable	PC1	PC2	PC3
Exchangeable K^+	0.233	−0.136	0.572
Exchangeable Na^+	−0.265	−0.204	0.236
NH_4^+-N	0.310	0.268	−0.224
NO_3^--N	−0.331	0.013	−0.137
Alkali-hydrolyzable nitrogen	0.235	0.163	0.187
Soil organic matter	0.242	0.489	0.047
Available phosphorus	0.215	−0.077	−0.643
Available potassium	0.305	−0.184	−0.241
Maize grain yield	0.157	0.597	0.052

Table S6. Pearson correlation matrix among soil properties and maize grain yield included in the principal component analysis.

Variable	pH	pHBC	Ex-K^+	Ex-Na^+	Ex-Ca^{2+}	Ex-Mg^{2+}	NH_4^+-N	NO_3^--N	AHN	SOM	Avail. P	Avail. K	Yield
pH	1.00												
pHBC	0.53**	1.00											
Ex-K^+	0.54**	0.34*	1.00										
Ex- Na^+	−0.54**	−0.32	−0.16	1.00									
Ex-Ca^{2+}	0.90**	0.52**	0.60**	−0.53**	1.00								
Ex- Mg^{2+}	0.82**	0.47**	0.62**	−0.52**	0.92**	1.00							
NH_4^+-N	0.58**	0.32	0.51**	−0.52**	0.56**	0.53**	1.00						
NO_3^--N	−0.70**	−0.45**	−0.55**	0.43**	−0.70**	−0.74**	−0.80**	1.00					
AHN	0.40*	0.16	0.25	−0.39*	0.39*	0.39*	0.52**	−0.47**	1.00				
SOM	0.38*	0.22	0.21	−0.59**	0.31	0.38*	0.74**	−0.48**	0.42**	1.00			
Avail. P	0.39*	0.18	−0.04	−0.43**	0.49**	0.59**	0.26	−0.42*	0.34*	0.23	1.00		
Avail. K	0.79**	0.37*	0.40*	−0.43**	0.71**	0.69**	0.44**	−0.60**	0.57**	0.27	0.58**	1.00	
Yield	0.05	−0.02	0.18	−0.47**	0.06	0.18	0.57**	−0.30	0.35*	0.79**	0.13	0.13	1.00

Note: Values are Pearson correlation coefficients calculated using the 36 plot-level observations included in the principal component analysis. * and ** indicate significant correlations at $P < 0.05$ and $P < 0.01$, respectively. pHBC, soil pH buffering capacity; Ex-, exchangeable; AHN, alkali-hydrolyzable nitrogen; SOM, soil organic matter; Avail. P, available phosphorus; Avail. K, available potassium.

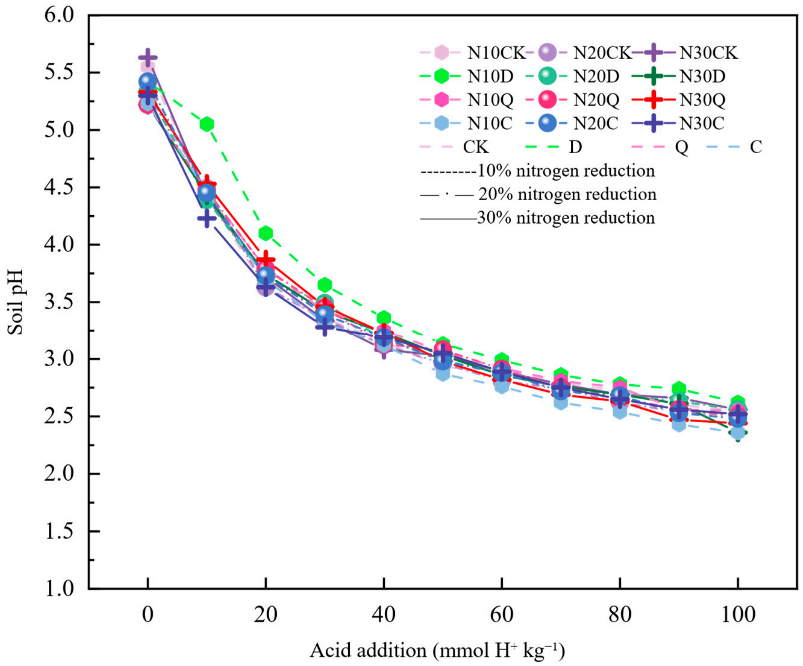

Figure S1. Full acid-titration responses of soils under nitrogen reduction and calcium-based amendment treatments. Symbols represent individual observations across the full acid-addition range. N10, N20, and N30 denote 10%, 20%, and 30% nitrogen reduction, respectively; CK, D, Q, and C denote no amendment, dolomite, quicklime, and fused calcium–magnesium phosphate fertilizer, respectively.

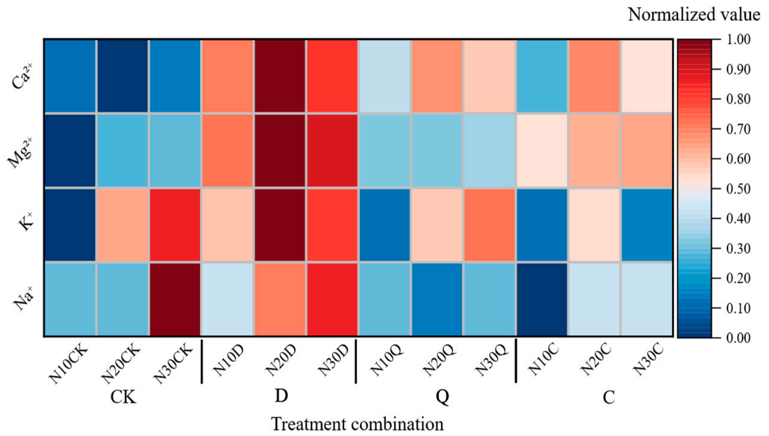

Figure S2. Heatmap of normalized exchangeable base-cation concentrations under different nitrogen reduction and calcium-based amendment treatments. Mean concentrations of exchangeable Ca^{2+}, Mg^{2+}, K^{+}, and Na^{+} for each treatment combination

were normalized separately for each ion using Normalized value. N10, N20, and N30 represent 10%, 20%, and 30% reductions in nitrogen application, respectively; CK, D, Q, and C represent no amendment, dolomite, quicklime, and fused calcium–magnesium phosphate fertilizer, respectively.
